# Understanding the Pyrimethamine Drug Resistance Mechanism via Combined Molecular Dynamics and Dynamic Residue Network Analysis

**DOI:** 10.3390/molecules25040904

**Published:** 2020-02-18

**Authors:** Arnold Amusengeri, Rolland Bantar Tata, Özlem Tastan Bishop

**Affiliations:** Research Unit in Bioinformatics (RUBi), Department of Biochemistry and Microbiology, Rhodes University, Grahamstown 6140, South Africa; g16a7782@campus.ru.ac.za (A.A.); g18t9691@campus.ru.ac.za (R.B.T.)

**Keywords:** malaria, drug resistance, dihydrofolate reductase, pyrimethamine, dynamic residue network, MD-TASK

## Abstract

In this era of precision medicine, insights into the resistance mechanism of drugs are integral for the development of potent therapeutics. Here, we sought to understand the contribution of four point mutations (N51I, C59R, S108N, and I164L) within the active site of the malaria parasite enzyme dihydrofolate reductase (DHFR) towards the resistance of the antimalarial drug pyrimethamine. Homology modeling was used to obtain full-length models of wild type (WT) and mutant DHFR. Molecular docking was employed to dock pyrimethamine onto the generated structures. Subsequent all-atom molecular dynamics (MD) simulations and binding free-energy computations highlighted that pyrimethamine’s stability and affinity inversely relates to the number of mutations within its binding site and, hence, resistance severity. Generally, mutations led to reduced binding affinity to pyrimethamine and increased conformational plasticity of DHFR. Next, dynamic residue network analysis (DRN) was applied to determine the impact of mutations and pyrimethamine binding on communication dispositions of DHFR residues. DRN revealed residues with distinctive communication profiles, distinguishing WT from drug-resistant mutants as well as pyrimethamine-bound from pyrimethamine-free models. Our results provide a new perspective on the understanding of mutation-induced drug resistance.

## 1. Introduction

Human malaria is a disease of global public health importance. Among the five causative Plasmodium species, *Plasmodium falciparum* is the most devastating [1]. The parasite is responsible for the highest share of the disease burden in sub-Saharan Africa, where it accounts for over 90% of malaria-related morbidity and mortality [2]. The prevalence and severity of clinical malaria in the endemic areas of this region are higher in pregnant women and in children below the age of 10 years [3,4]. Scientific reports highlighting the efficacy of the antimalarial drug combination, sulphadoxine pyrimethamine (SP), in intermittent preventive treatment during pregnancy (IPTp) and seasonal malaria chemoprevention (SMC) in children [4,5,6] led to the current WHO recommendations of its usage for IPTp and SMC in children. 

**Figure 1:**
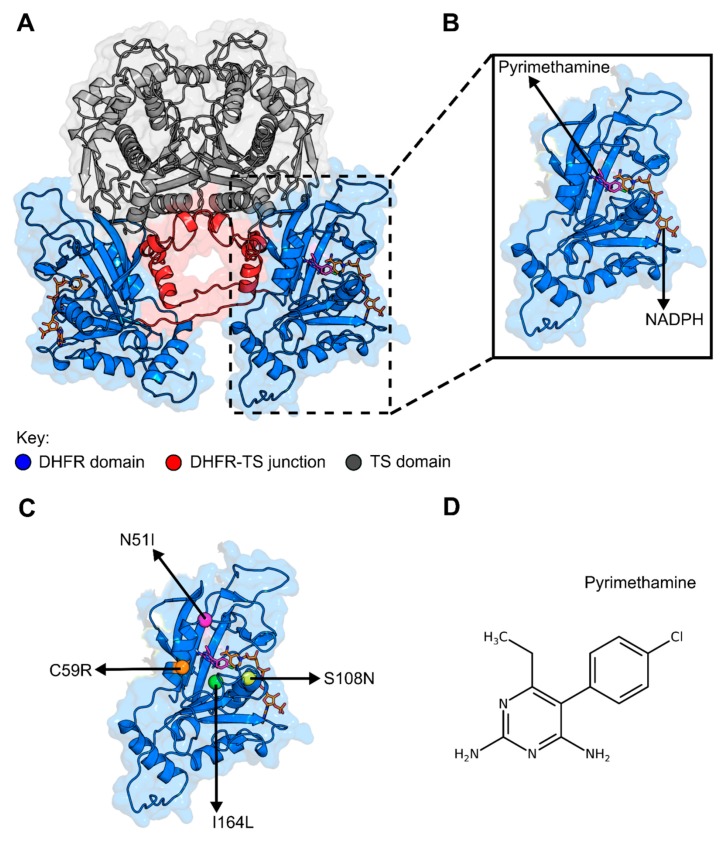
(**A**) Illustration of the *P. falciparum* thymidylate synthase domain of dihydrofolate reductase (DHFR-TS) dimeric assembly: The structure was generated using homology modeling technique. *P. falciparum* Protein Data Bank (PDB) ID: 3QGT was used as a template. Color key: blue: DHFR domain, red: DHFR-TS junction, grey: TS domain. (**B**) Zoomed in image of the DHFR domain complexed with nicotinamide adenine dinucleotide phosphate (NADPH) cofactor and pyrimethamine. (**C**) Structural mapping of pyrimethamine-resistant mutations assessed in this study. (**D**) Wireframe representation of the structure of pyrimethamine.

The pyrimethamine component of SP is an antifolate and a selective inhibitor of *P. falciparum* dihydrofolate reductase (*Pf*DHFR). *Pf*DHFR is among the best clinically validated and well-defined targets for antimalarial drug discovery [7]. Structurally, it forms part of a dimeric assembly consisting of monomeric bifunctional units (Figure 1A,B). Each monomer is 608 residues long and is made up of a DHFR subunit (231 residues long N-terminal domain) and a thymidylate synthase (TS) subunit (288 residues C-terminal domain) joined by an 89-residue junction region [8]. DHFR functions by catalyzing the reduction of 7,8-dihydrofolate (DHF) to 5,6,7,8-tetrahydrofolate (THF), using the reduced form of nicotinamide adenine dinucleotide phosphate (NADPH) as cofactor [9]. It thus plays a key role in the folate biosynthetic pathway and generation of the DNA base, deoxythymidine monophosphate (dTMP), hence its indispensability in parasite replication [9,10,11]. 

While the TS domain of *Pf*DHFR-TS shares considerably high sequence identity across various species, including humans [8], the DHFR domain is relatively divergent. Sequence differences, particularly around the active site region, have allowed for the development and use of species-specific antifolates such as pyrimethamine and cycloguanil [8]. However, application of currently approved drugs against this target is being impaired by the development and spread of drug resistance [12]. Generally, drug resistance in *Plasmodium* parasites is associated with either point mutations or copy number variations in related genes [13], which results in either impaired drug uptake by the parasite, parasite efflux of the drug from target site, disruption in mitochondrial membrane potential, or steric hindrance to drug binding within the parasite enzyme target [13,14].

In this work, we focus on resistance to pyrimethamine which is mediated by non-synonymous mutations in the *DHFR* gene of *P. falciparum* [15]. Previous reports indicate that the mechanism of resistance is based on steric constraints to pyrimethamine binding and to changes in the main chain configuration of *Pf*DHFR, both caused by mutations involving residues with bulky side groups in the active site region of the enzyme, with exacerbated effects as mutations accumulate [8,12,13]. Accordingly, this compromises the use of SP and is further aggravated by the drug pressure resulting from continuous usage [7,9,10,13].

Several experimental studies have demonstrated various mutations that are responsible for pyrimethamine and other antifolate drug resistance [14,15,16,17,18,19]. However, only mutations at four loci 51, 59, 108, and 164 (Figure 1C) have been associated with pyrimethamine resistance from field isolates [16]. Lone mutation at codon 108 (S108N) (single mutant (SM)), which is the least resistant mutant found in nature, was shown to be the genesis for all pyrimethamine-resistant mutations [15]. Stepwise mutations at other loci resulted in moderately resistant double mutants N51I+S108N (DM1) and C59R+S108N (DM2), high resistant triple mutants N51I+C59R+S108N (TM1) and C59R+S108N+I164L (TM2), and highly resistant quadruple mutant N51I+C59R+S108N+I164L (QM) [15]. While resistance is seen to increase with added mutations, this is not the case with the catalytic efficiency of the enzymes [15]; SM and DM1 share similar enzymatic kinetics with the WT enzyme, QM possesses relatively impaired enzymatic kinetics [15], while DM2, TM1, and TM2 are highly compromised [15]. X-ray crystallography and molecular modeling experiments have provided valuable information on resistance-specific protein–ligand interactions [13]. Primarily, limited molecular dynamics (MD) simulation studies have leveraged the understanding of system stabilities in the presence or absence of mutations [13,18,19]. These have however not included all the relevant mutations, and analyses have hardly explained function-related essential motions and intra-protein residue communication dynamics, which provide further insights into the understanding of protein behavior.

In this work, we sought to understand the effects of mutations (described above) in the active site of DHFR domain, responsible for resistance to pyrimethamine. We employed comparative modeling techniques to build full-length 3D structures of both wild type (WT) and clinically relevant mutant DHFR proteins. Using molecular docking, pyrimethamine was docked to each of the proteins. This revealed differences in binding poses which apparently translates to unfavorable binding affinities in some of the mutants. All holo and DHFR-pyrimethamine complexes were subjected to 100-ns all-atom MD simulations. Post-MD analysis revealed that overall structural folds of DHFR remain relatively preserved in the presence of mutations and pyrimethamine. However, mutations moderately modulate pyrimethamine-complexed DHFR conformations along with innate structural flexibility, thus weakening pyrimethamine binding affinity. For the first time, we applied DRN analysis on these systems to reveal critical communication patterns underlying resistance. We found that active site and inter-domain residues are, natively, high traffic centers. These residues show varied adjustments to mutations or pyrimethamine binding in different systems. Altogether, this work depicts mutation-induced changes in *P*fDHFR that have resulted in pyrimethamine resistance: highlighting conformational, energetic, and residue communication differences.

## 2. Results and Discussion

### 2.1. Pyrimethamine Docked Differently to Protein with Resistance Mutations due to the Changes in the Active Site

Although the DHFR domain (Met1-Asn231) was considered in this study, the entire DHFR-TS dimeric assembly was modeled (Figure 1A) with slight modifications as outlined in the methodology. Multiple sequence alignments failed to identify suitable templates for most of the missing residues which constituted predominantly loop regions. This however is not surprising as the *Plasmodium* genome possesses unique sequences which account for up to two-thirds of its rather distinctive proteome [20]. The missing residues in the DHFR domain were modeled as an entire loop while only 9 out of the 51 missing residues in the junction region (linked to the N-terminal of the TS domain) were included in the model. This was done to allow for the critical length necessary for TS activity in the dimer [21]. Top models passing assessments by all applied evaluation metrics were considered for this study (Table S1). These models were trimmed to obtain the DHFR domain and further prepared for molecular docking. 

Docking validation in WT resulted in similar docking orientations (RMSD = 0.66 Å) and interactions, relative to the crystal structure (PDB ID: 3QGT). The final docking experiment produced complexes with slight differences in docking scores (Table S2). Further analysis revealed differences in binding poses (Table S2 and Figure S1) especially for DM1 and TM2 in which pyrimethamine bound with its 4-chlorophenyl group oriented towards the interior of the active site (Table S1). The observed differences in binding affinity/poses are most likely due to induced changes within the active site caused by mutations. Apart from the induced steric clash to pyrimethamine binding caused primarily by S108N mutation, the N51I and I164L mutations are known to induce an increase in the active site size [13], leading to low binding affinity for small inhibitors such as pyrimethamine. This could also explain the change in orientation of pyrimethamine in these mutants DM1 and DM2.

### 2.2. Global Analysis Revealed Differences in the Conformational Spaces Between WT and Proteins with Resistance Mutations in the Absence and Presence of the Drug

Although highly effective, molecular docking disregards various degrees of protein flexibility, along with practical microenvironment conditions important for rational protein-function studies. For this reason, MD simulations were implemented to comprehensively understand systemic effects of resistance mutations. All-atom 100-ns MD simulations were performed for WT and six mutated DHFR systems either in apo form or complexed with pyrimethamine, totaling fourteen runs as described in the Methodology section. To understand the effect of mutations in the absence and presence of ligands to the global protein motions, the protein backbone root mean square deviation (RMSD), radius of gyration (*Rg*), principal component analysis (PCA), and the binding free energy (BFE) were calculated for each system and compared.

#### 2.2.1. RMSD Analysis

RMSD values of protein backbone atom coordinates were computed to assess conformational evolution of DHFR with reference to the starting structure (Figure S2). RMSD values converged within ~0.35 nm (Table S3), suggesting that the overall structural folds were relatively preserved. Besides RMSD values of S108N, N51I_C59R_S108N, and C59R_S108N_I164L-PYR which drift initially before plateauing, RMSD differences between WT and mutant systems (both pyrimethamine-bound and pyrimethamine-free) were mostly minimal. 

**Figure 2:**
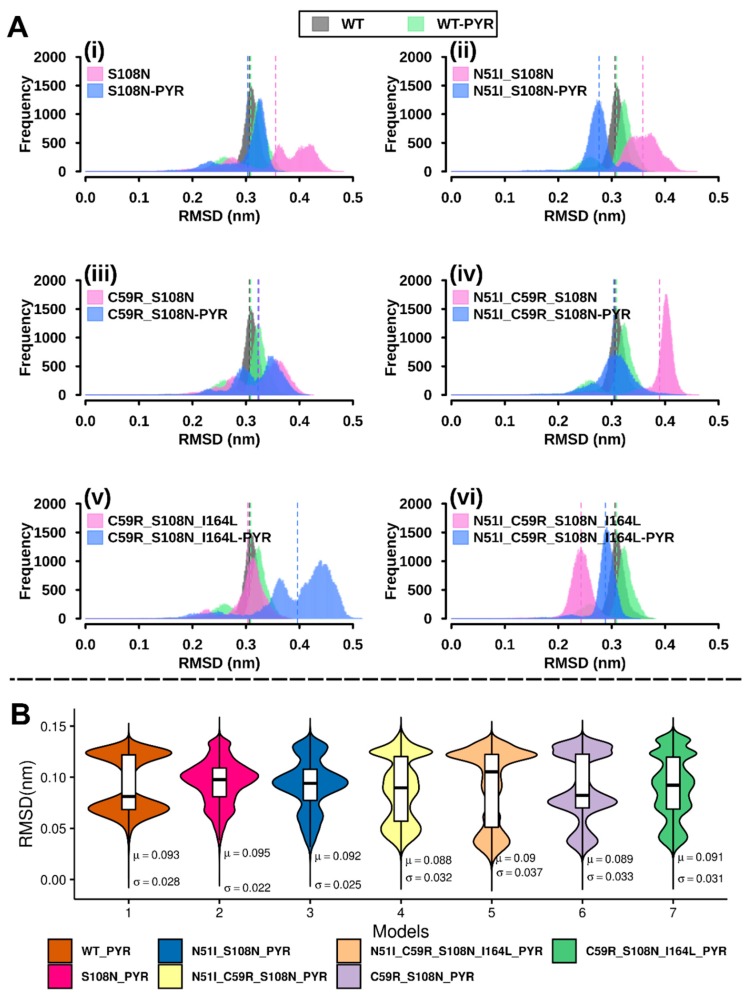
(**A**) Histograms showing protein root mean square deviation (RMSD) frequency distribution during 100-ns simulations: RMSD values were computed based on back-bone atom positions. The width corresponds to the number of conformations sampled by proteins over the molecular dynamics (MD) simulation. Y-axis (frequency) represents number of times a specific conformation was sampled during the MD simulation. (**i**) S108N, (**ii**) N51I_S108N, (**iii**) C59R_S108N, (**iv**) N51I_C59R_S108N, (**v**) C59R_S108N_I164L, and (**vi**) N51I_C59R_S108N_I164L. Color key: black WT—holo, green WT—pyrimethamine complex, magenta mutant—holo, and blue mutant—pyrimethamine complex. (**B**) Kernel density estimation graphs overlaid with boxplots showing the distribution of ligand RMSDs for each complex. Density traces were plotted symmetrically on each side: the width corresponds to the frequency of RMSD occurrence. Boxplots highlight the first, second (median), and third quartiles. The mean and standard deviation values are indicated by µ and σ, respectively.

To be able to observe potential discrete conformational changes in protein systems and hence to understand the variation effect on them, RMSD distribution histograms of the WT and variant proteins both in ligand free and ligand bound were generated, as previously applied [22,23,24] (Figure 2A). The pyrimethamine-free WT model displayed nearly normal RMSD distribution while the bound system showed a bimodal curve, suggesting that the former predominantly sampled a single conformer while the latter sampled at least two conformations during simulation. Compared to the WT and WT-PYR, pyrimethamine-free mutated systems S108N, N51I_S108N, C59R_S108N, and N51I_C59R_S108N_I164L and pyrimethamine-bound mutated systems S108N-PYR, N51I_S108N-PYR, C59R_S108N-PYR, and C59R_S108N_I164L displayed multimodal RMSD distributions, indicating that mutations destabilize the systems generating multiple equilibrium states during simulation.

Further, ligand RMSD calculations were conducted to assess how much pyrimethamine’s binding conformation adjusted over time. Periodic jumps in RMSD values around 0.08 nm were observed across all models (Figure S3). Density plots of pyrimethamine RMSDs (Figure 2B) show the ligand samples’ multiple equilibrium states during 100-ns simulation. The WT-PYR model displays a symmetric bimodal RMSD distribution. This observation is attributed to conformational isomerism exhibited during simulation, whereby the 4-chlorophenyl group (Figure 1D) oscillates about the rotatable phenyl-pyrimidine bond (Video S1). Mutated models show multimodal (of more than two peaks) ligand RMSD distribution, suggesting that mutations destabilize protein–ligand interactions. Markedly, RMSD values of the pyrimethamine bound to the highly resistant QM (N51I_C59R_S108N_I164L) were largely spread out and recorded the largest standard deviation value (0.037 nm) (Figure 2B), suggesting a highly mobile and unstable ligand. This hints that increasing the number of mutations could engender significant detrimental effects on protein–ligand affinity, in agreement with previous observations from in vivo and in vitro experiments [15].

#### 2.2.2. RMSF Analysis

**Figure 3:**
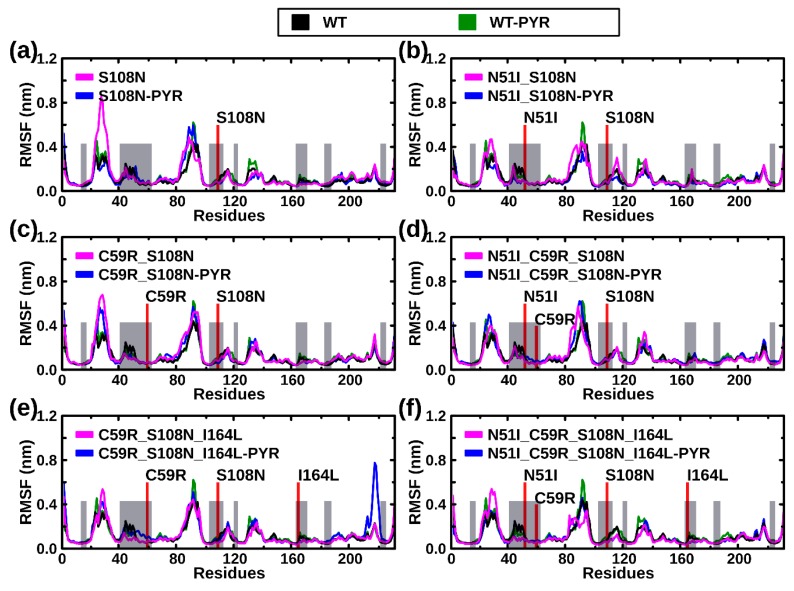
Average per residue root mean square fluctuation (RMSF) computed from Cα atoms: (**a**) S108N, (**b**) N51I_S108N, (**c**) C59R_S108N, (**d**) N51I_C59R_S108N, (**e**) C59R_S108N_I164L, and (**f**) N51I_C59R_S108N_I164L. Color key: black: WT holo, green: WT complexed with pyrimethamine, magenta: mutant holo, blue: mutant complexed with pyrimethamine.

To monitor residue fluctuations, the root mean square fluctuation (RMSF) of Cα atoms was calculated. In general, most residues located at flexible loop regions (residues 20–40, 85–100, and 130–140) yielded uppermost fluctuations (Figure 3); both pyrimethamine-free and pyrimethamine-bound mutated systems exhibit enhanced and irregular fluctuation patterns in these regions. Although comparisons of RMSF profiles between WT and mutants largely show inconsiderable global differences, meaning invariably stable systems in the presence and absence of mutations or the ligand (pyrimethamine), residues 210–225 of the C59R_S108N_I164L-PYR complex uniquely exhibited larger RMSF values.

#### 2.2.3. Rg Analysis

The radius of gyration (*Rg*) provides useful information about spatial packing of atoms relative to the center of mass of a protein. Besides C59R_S108N, C51I_C59R_S108N, and N51I_C59R_S108N_I164L-PYR which depict more compact systems (Figure S4), *Rg* profiles of both wild type and mutated models generally registered inconsiderable differences, with the *Rg* values converging within the range of 1.85–1.90 nm. Assessment of relative *Rg* frequencies revealed different bimodal distributions in C59R_S108N, N51I_C59R_S108N, and N51I_C59R_S108N_I164L (pyrimethamine-free) and in S108N-PYR (pyrimethamine-bound) relative to the wild type systems (WT and WT-PYR), suggesting that the associated mutations induce substantial adjustments in spatial packing of respective systems (Figure S5).

#### 2.2.4. Mutations Moderately Modulated Conformational Dynamics

To understand the impact of mutations on conformation redistribution in DHFR, essential dynamics was performed on the protein backbone atoms. Realignment of conformation ensembles can be correlated to a proteins’ gain/loss of function [25]. Considering the fact that the mutations studied here occur at the active site, they are bound to influence protein recognition and hence binding behaviour. Principal component analysis (PCA) enables the extraction of large-scale important motions that occur during simulation by reducing the dimensionality of the conformational space. To retain as much information as possible, the top two eigenvectors (PC1 and PC2), from a total of 2079, corresponding to eigenvalues possessing the largest percentage variance (Table S4) were selected for interpretation. Also, the trace values, which correspond to the sum of eigenvalues (total variance), associated with the transformation matrix and which can highlight the overall intrinsic protein flexibility, were noted (Table S5). Two-dimensional projections of these eigenvectors (PC1 and PC2) show that the presence of either/both pyrimethamine and mutations yield different patterns of motion, albeit within a restrained space as evidenced by small trace values (Figure S6 and Table S5). 

While 2D projections offer a general outlook of clusters formed and the accessible conformational space explored during simulation, the free energy landscape provides a perspective view of the transition subspace along with conformer associated abundance. 

**Figure 4:**
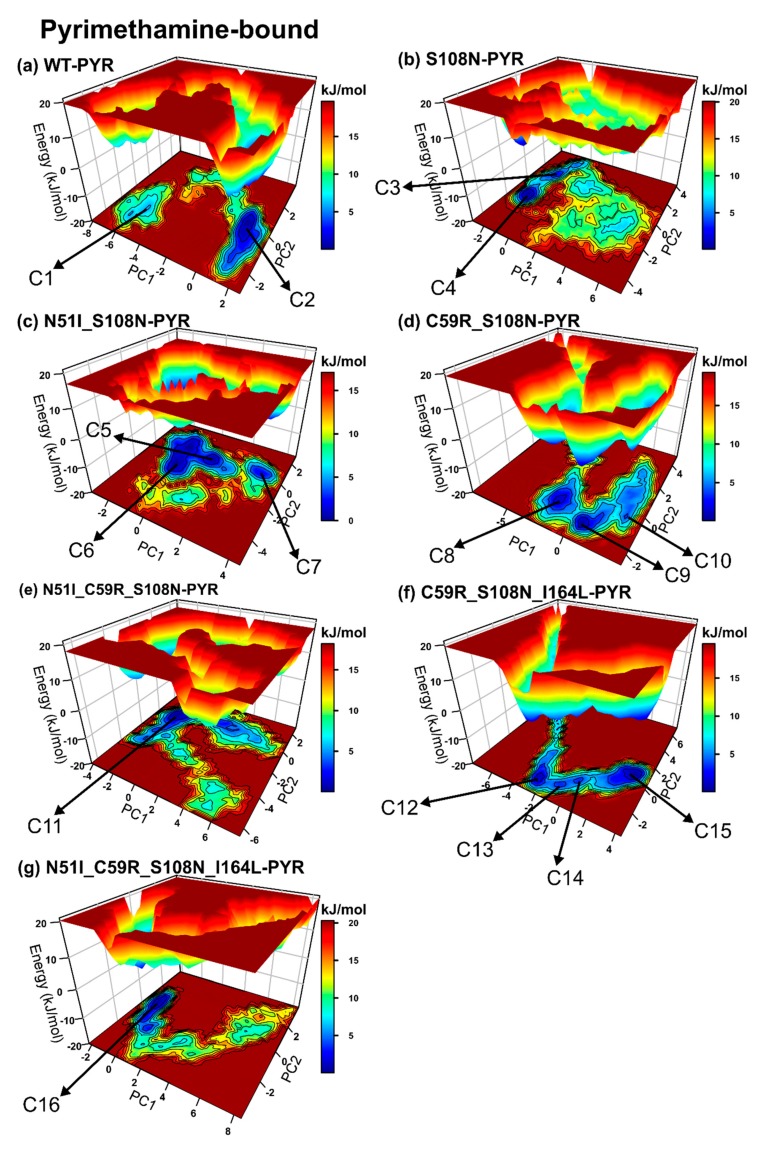
Free-energy landscape plots of pyrimethamine-bound systems: Perspective view of free-energy landscape as a function of the first two principle components (PC1 and PC2). The landscapes were color coded from maroon (energy maxima) to blue (energy minima). Conformations sampled are labeled C1–C16.

**Figure 5:**
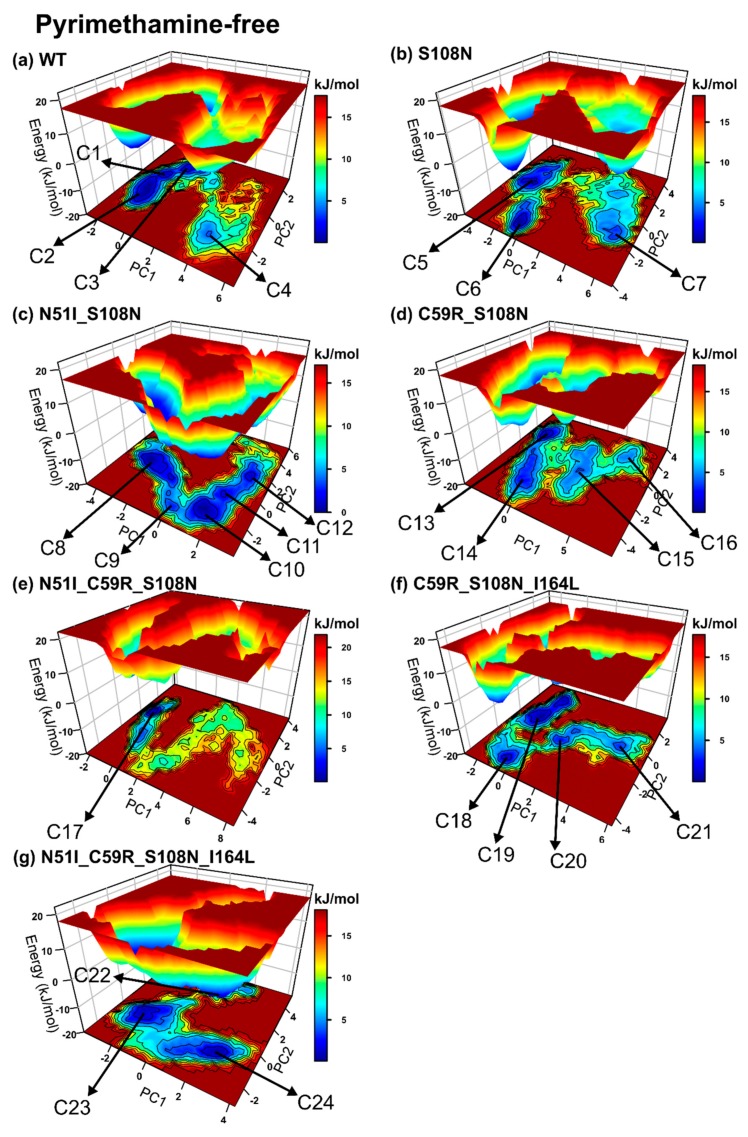
Free-energy landscape plots of pyrimethamine-free systems: The coloring scheme used in Figure 4 was employed here as well. Conformers sampled during simulation are labeled C1–C24.

We evaluated the effects of mutations on the simulated systems: (1) Regarding pyrimethamine-bound complexes, the WT-PYR and N51I_C58R_S108N_I164L-PYR display nearly similar population shift patterns, while S108N, C59R_S108N-PYR, N51I_S108N-PYR, N51I_C59R_S108N-PYR, and C59R_S108N_I164L-PYR displayed reverse patterns during simulation (Figure S6). The WT-PYR (two conformers), N51I_C59R_S108N-PYR, and N51I_C59R_S108N_I164L-PYR display conformation population shifts leading to a single dominant energy basin (single well energy landscapes), suggesting relatively rigid systems regardless of mutations (Figure 4). Contrarily, mutated complexes S108N-PYR (two conformers), N51I_S108N-PYR (three conformers), C59R_S108N-PYR (three conformers), and C59R_S108N_I164L-PYR (four conformers) constructed elongated valleys with a series of folding funnels connected by low-lying energy barriers. This suggests that the above mutations induce structural instability on the pyrimethamine-bound DHFR, resulting in conformational heterogeneity. S108N-PYR spans a broader conformational space indicative of a highly flexible system. (2) Regarding pyrimethamine-free systems, we observe that the models generally display more rugged energy landscapes relative to pyrimethamine-bound models, indicating highly mobile systems. The WT, S108N, C59R_S108N, N51I_C59R_S108N, and N51I_C58R_S108N_I164L exhibit nearly similar patterns of ensemble shifts, while N51I_S108N and C58R_S108N_I164L show reverse patterns (Figure S6). Comparable to the wild type, all mutated systems except N51I_C59R_S108N visit multiple metastable conformers: WT: four conformers, S108N: three conformers, N51I_S108N: five conformers, C59R_S108N: four conformers, C58R_S108N_I164L: four conformers, and N51I_C59R_S108N_I164L: three conformers, (Figure 5) suggesting that the systems possess inherent flexibility that is likely unrelated to generated mutations. N51I_C59R_S108N exhibits a single-well folding route, indicating that the presence of mutations confers structural rigidity to some degree. 

Collectively, all mutated pyrimethamine-free models recorded higher trace values relative to the WT-PYR (Table S3), meaning that the presence of mutations enhances structural flexibility and could accordingly modulate ligand-binding events. On the other hand, only the C59R_S108N-PYR, N51I_C59R_S108N-PYR, and C58R_S108N_I164L-PYR models of pyrimethamine-bound simulations recorded higher trace values compared to WT-PYR, suggesting mutation-specific influence on structural mobility.

Next, we assessed the effects of pyrimethamine-binding on conformation redistribution by cross-comparing pyrimethamine-free versus bound models. As stated earlier, the pyrimethamine-free systems generally exhibit rugged energy landscapes characterised by multiple metastable states. Besides S108N-PYR, pyrimethamine-bound systems generally construct narrow energy basins with deeper funnels, showing that the presence of pyrimethamine reasonably dictates rigidity to the structures. Relative to pyrimethamine-free models, the majority of pyrimethamine-bound models, including S108N-PYR, N51I_S108N-PYR, C59R_S108N-PYR, and N51I_C59R_S108N_I164L-PYR, possess lower trace values, implying improved structural rigidity. 

It was increasingly clear that DM1 (N51_S108N), DM2 (C59R_S108N), and TM2 (C59R_S108N_I164L) enhance conformational heterogeneity while TM1 disfavours conformational plasticity. Altogether, mutations moderately modulate pyrimethamine-bound DHFR conformations along with innate structural flexibility and, as such, could affect pyrimethamine’s association constants.

#### 2.2.5. Mutations Weakened the Binding Affinity of Pyrimethamine to DHFR

To investigate the influence of mutations on the solidity of protein–ligand interactions, binding free-energy calculations were performed on pyrimethamine-bound complexes using the Molecular Mechanics Poisson−Boltzmann Surface Area (MM/PBSA) method [26]. It was discovered that the total binding free-energy values across all mutated systems were higher, meaning pyrimethamine binding was more favored in the wild type than it was in mutated DHFR (Table 1). Relative to the wild type (−127.20 ± 0.21 kJ mol^−1^), we observed a general increasing trend in total binding free-energy values with increase in number of mutations, in agreement with findings of previous studies [15]. 

Mutated complexes N51I_C59R_S108N_I164L (−84.90 ± 0.25 kJ mol^−1^) and C59R_S108N (−84.40 ± 0.23 kJ mol^−1^) yielded the highest total binding energies. The van der Waals energy term ($\Delta E_{\mathrm{vdW}}$) contributed the most to the total binding free energy. Also, comparing the wild type with mutated systems, $\Delta E_{\mathrm{vdW}}$ was majorly responsible for increase in binding free energy in mutants. Per residue, decomposition calculations revealed that residues ASP54 (8.32 kJ mol^−1^), LEU46 (−5.82 kJ mol^−1^), PHE58 (−12.33 kJ mol^−1^), and ILE164 (−5.35 kJ mol^−1^) contributed substantially to the total binding free energy in the wild type model (Figure S7 and Table S6). The above residues are distributed within the pyrimethamine binding region; hence, they play an important role in the affinity and stability of protein–ligand interactions. In comparison to the wild type, residues 164, 54, 55, 15, 112, and 116 predominantly displayed considerable variations in total binding free energy across all mutated systems (Table S6). Accordingly, these residues could play a vital role in shifting protein–ligand dynamics after mutation.

Assessment of intermolecular hydrogen bond numbers between DHFR and pyrimethamine showed that both wild type and mutated systems possess at least a single hydrogen bond during simulation. Besides N51I_S108N, C59R_S108N, and C58R_S108N_I164L which registered inconsistent numbers, all models consistently recorded 2–4 hydrogen bond numbers (Figure S8).

Various experimental reports suggest that antifolates, including pyrimethamine, adopt wild type/mutant-specific association states with DHFR. For instance, Cocco and coworkers [27] demonstrated that pyrimethamine, trimethoprim, and methotrexate preferably bind WT DHFR with a protonated N1 position of the pyrimidine ring under physiological conditions. Abdizadeh et al. [28] showed that trimethoprim preferably associates with the *Escherichia coli* DHFR mutant D27N in its neutral state whereas both its neutral and protonated states equally bind the D27S mutant. It should be pointed out that the results discussed here illustrate the binding behavior of pyrimethamine in its neutral state to both wild type and mutated models. While the effects of pyrimethamine’s protonation state on its binding affinity are likewise important, these conditions were not incorporated in the objectives of this study. Moreover, the binding states of the drug to mutated *Pf*DHFR systems examined here are yet to be established.

### 2.3. Differences in Intra-Protein Communication Patterns due to Mutations and Ligand Binding were Observed

#### 2.3.1. Dynamic Residue Network Analysis

**Figure 6:**
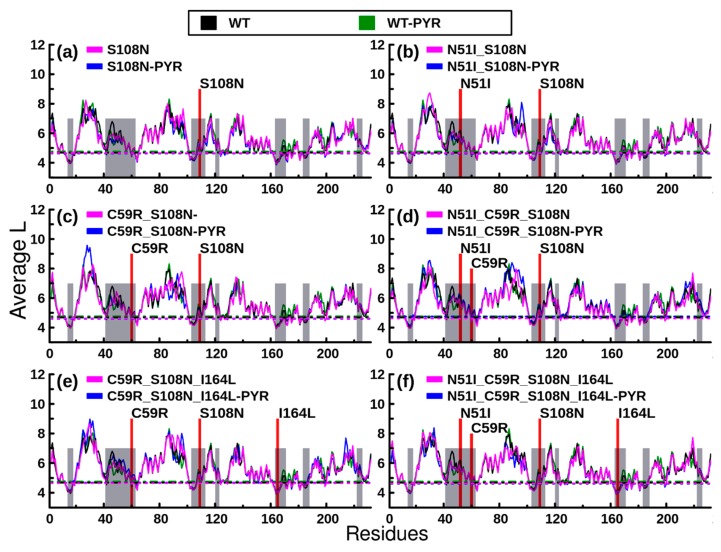
Dynamic residue network analysis: Average shortest path (*L*) results. Color key: black: WT holo, green: WT complexed with pyrimethamine, magenta: mutant holo, blue: mutant complexed with pyrimethamine. Shaded areas are zones of protein–ligand interactions. Lower threshold values are indicated by respective color-coded, dotted lines. Shaded areas are regions of ligand interaction within the active site.

Residue interaction network (RIN) analysis of a protein backbone is useful in identifying key residues involved in intra-protein communication. This can be used to explore differences in information flow between wild type and mutant as well as ligand-bound and ligand-free systems [29,30]. Two fundamental network properties, average shortest path (*L*) and *betweenness centrality* (*BC*), have proven useful in RIN analysis [31,32]. *L* denotes the reachability of a residue by all other residues in a communication network, while *BC* highlights how frequent a residue participates in the shortest paths between all residue pairs. Both metrics accentuate residue importance in protein communication. The *L* and *BC* measures become even more robust when computed as running averages across an MD trajectory, in what is known as dynamic residue network (DRN) analysis [23,24,30,32]. We present per residue reachability as plots of average *L* in (Figure 6). Troughs in average *L* plots represent residues with low average *L* values and hence higher reachability regions. Generally, the distribution of average *L* troughs across all models (both pyrimethamine bound and unbound) was similar; the profiles registered pairwise correlation values of 0.90 and above. Markedly, residues within or close to pyrimethamine/substrate-binding sites (grey shaded in Figure 6) recorded low average *L* values. This observation corroborates findings of our previous study associating regions possessing low average *L* values with residue communities located within the active site (substrate binding site) [30]. Generally, residues 12–16, 18, 59, 63, 101–105, 123, 161–166, 170–171, 174–175, 180, 181, and 183–185 registered significant low average *L* values across all systems. In regard to pyrimethamine-free models, Pro198 identified across all mutated systems was conspicuously absent in the WT model. On the contrary, Cys17 and Gly41 were uniquely identified in the WT model. It is likely that local adjustments of residue accessibility involving the above molecular fingerprints determine the functional separation between a native (WT) and nonnative (mutated) system. On the other hand, pyrimethamine-bound models displayed insignificant variability in residue accessibility following pyrimethamine binding. 

**Figure 7:**
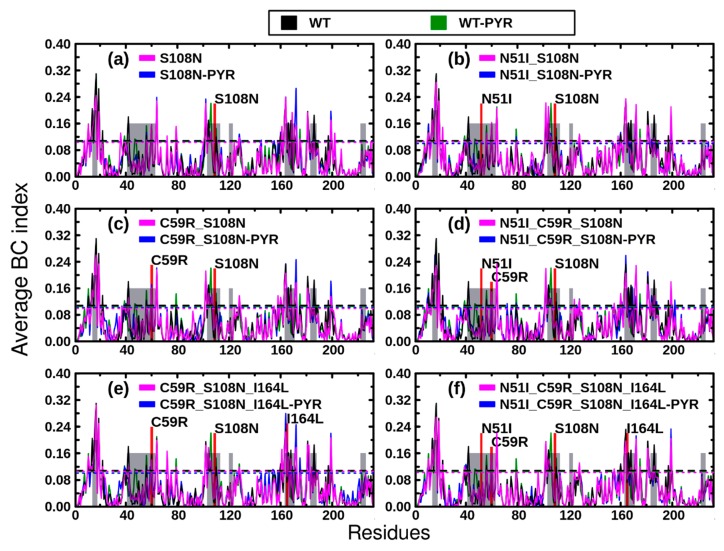
Contact network analysis: *Betweenness centrality* (*BC*) results. Color key: black: WT holo, green: WT complexed with pyrimethamine, magenta: mutant holo, blue: mutant complexed with pyrimethamine. Lower threshold values are indicated by respective color-coded, doted lines. Grey shaded areas are regions of ligand interaction within the active site.

To further understand the influence of mutations and pyrimethamine binding on intra-protein communication, average *BC* indices were calculated (Figure 7). Peaks of average *BC* plots depict residues with high *BC* values and hence high communication centers. Since *BC* values were more centered around the mean, we set a threshold value at plus two standard deviations to identify residues with high *BC* indices (substantial peaks). Overall, regions showing significantly high *BC* values were located within or close to substrate/ligand-binding sites (shaded grey in Figure 7). These results recapitulate previous findings reporting that active sites of proteins possess high and low average *BC* and average *L* values, respectively [23,30,33]. 

Using WT holoenzyme as reference, twenty-four key communication residues were identified: 10, 13, 15, 16, 18, 21, 41, 55, 59, 63, 101, 103, 105, 109, 159, 162, 163, 165, 167, 170, 180, 181, 185, and 196. Besides residues 41 and 196, all the above can be grouped into five different communication hubs: α: 10–21, β: 55–63, γ: 101–109, δ: 159–170, and ε: 180–185. Interestingly, these hubs are located in distinct secondary structures spanning the active site region (Figure S9). Residues 41 and 196 are situated in loops outside the active site. Some residues within the identified hubs, Asp10, Tyr12, Ile14, Cys15, Ala16, Met55, Lys181, and Thr185, correspond to residues previously identified to be important in DHFR activity [34], including involvement in either interdomain contacts or interactions with dihydrofolate, antifolate inhibitors, or the NADPH cofactor. Among these residues, Ala16, which is highly conserved and involved in important interactions with NADPH [34], showed the highest *BC* value in all systems except in TM2; Ile163 (situated hub δ and of which the functional information is unknown) was rather the most important residue for communication. This is likely reflective of global shifts in residue communication patterns within the TM2 mutant and might be responsible for its highly impaired catalytic efficiency compared to other mutants as has been demonstrated in in vitro studies [15]. Other residues, including Cys15, Met55, and Ile164, contribute substantially to total binding free energy of pyrimethamine in the wild type enzyme. Intriguingly, some of the residues possessing high *BC* values, 10–14, 57–63, 159–162, and 180–184, form part of the positively charged groove at the back of the active site responsible for critical interactions (with the negatively charged junction region helix 285–294) necessary for *Pf*DHFR-TS protein folding and function [21,34,35]. These findings are in agreement with previous studies which found high centrality residues around active site and linker interphases of protein domains [23,30,33].

While deleterious mutations, such as cancer-causing mutations, mainly target residues with high centrality [36], the only resistance-related residue possessing high *BC* values in the WT *Pf*DHFR was Cys59. Cys59 is mutated to arginine in DM2, TM1, TM2, and QM. The above mutants have previously demonstrated poor catalytic properties in vivo [15]. It is thought that the mutation at Cys59 is compelled to occur in the context of other mutations to help improve substrate binding affinity by binding the glutamyl moiety of the *Pf*DHFR substrate dihydrofolate [35]. 

Finally, pairwise Pearson’s correlation of the raw average *BC* values revealed strong positive correlations (r = 0.83–0.92) among all pyrimethamine-free systems. This indicates that there were only subtle differences in average *BC* values across the systems [37], which can be expected since drug-resistance mutations evolve in a conservative fashion such that protein fitness is not overly jeopardized. To further elucidate mutation- and pyrimethamine-induced differences on protein communication, we calculated the changes in *BC* (Δ*BC*) values as differences between WT and mutants and as differences between ligand-free and corresponding ligand-bound systems, as demonstrated in previous studies [22,30].

#### 2.3.2. Mutation-Induced Changes in *Pf*DHFR Intra-Protein Communication did not Directly Relate to Pyrimethamine Resistance

**Figure 8:**
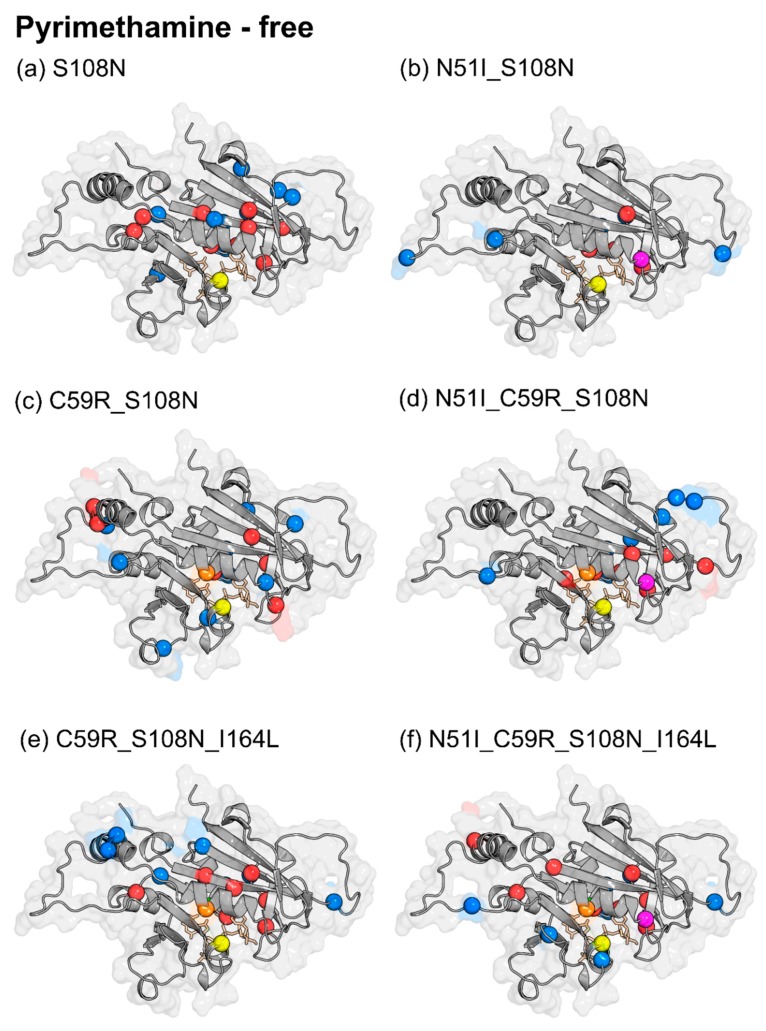
Effects of mutations on intra-protein communication: Structural mapping of residues showing significant changes in average *betweenness centrality (BC).* Changes in residue centrality (Δ*BC*) values were obtained by calculating WT-free less mutant-free average *BC* values. Residues with significant changes were obtained by using a cutoff value of +/−2 SD of Δ*BC* values for each system. Red spheres represent residues with positive Δ*BC* values, while blue spheres are residues with negative Δ*BC* values. Also shown in spheres are pyrimethamine-resistant point mutations studied here: Color key: magenta: N51I, orange: C59R, yellow: S108N, and green: I164L.

In order to assess mutation-induced effects on protein communication, Δ*BC* values were obtained by subtracting the mutant average *BC* values from WT as follows: pyrimethamine-free (WT-free less mutant-free) (Figure 8 and Figure S10) and pyrimethamine-bound (WT-pyrimethamine less mutant-pyrimethamine) (Figures S11 and S12). Table 2 shows the residues with significant changes in *BC*, obtained by applying a cutoff value of +/−2 SD for each mutant system, while Figure 8 and Figure S12 present the residues with significant Δ*BC* values mapped to the *Pf*DHFR structure. Residues with positive Δ*BC* values are colored in red while those with negative Δ*BC* values are colored blue. Positive Δ*BC* values denote a decrease in connectivity of the residue as a result of mutation, while negative Δ*BC* values denote increased connectivity.

Despite the underlying differences in the combinations of drug-resistance mutations, an interesting balance was noticed between the total number of residues with enhanced connectivity and those with diminished connectivity due to mutation in each system (Figure 8 and Figure S12). All systems had nearly equal number of residues with enhanced and diminished connectivity (Table 2). This might be reflective of a strict compensatory mechanism to global shifts in residue interaction networks, resulting from drug-resistance mutations. Such compensation could be required to salvage possible impairments in enzymatic fitness, imposed by mutations. Although native state (WT-free) high-centrality residues as well as drug-resistance mutation residues are mainly localized within the active site region, changes in residue connectivity due to drug-resistance mutations are both within and outside the active site (Figure 8 and Figure S12). This points to distant effects on residue connectivity induced by drug-resistance mutations. Similar long-range effects due to mutations have been reported in previous studies [22].

To check for any linear relationships among models with respect to Δ*BC*, an all versus all pairwise Pearson’s correlation was performed. As opposed to average *BC* values, pairwise correlation calculations of Δ*BC* values suggested more visible differences due to mutation in pyrimethamine-free (r = 0.44–0.73) and pyrimethamine-bound (r = 0.60–0.78) systems (Figure S13A,B,D). TM2 was revealed to be the most different (r = 0.44 – 0.60) in both the pyrimethamine-free and pyrimethamine-bound systems (r = 0.6–0.73). TM1 yielded similar correlation values with TM2 in the pyrimethamine-bound system. As mentioned earlier, in vitro experiments have shown that TM2 possesses the highest level of impaired catalytic efficiency followed by TM1 [15]. These findings suggest that apparent differences in residue communication due to drug-resistance mutations might form the basis for the experimentally observed differences in enzyme activity. Gln171 largely yielded negative substantial Δ*BC* values across all pyrimethamine-free mutants, suggesting mutation-specific enhancement of its connectivity.

A further look at pyrimethamine-bound Δ*BC* calculations revealed enhanced network connectivity for Ser167, Val168, and Tyr170. These residues together with Gln171 form part of the alpha helix responsible for stabilizing NADPH [34]. Considering NADPH’s proximity to pyrimethamine, chances are that derangement of information flow network involving the above residues could have cascading effects, destabilizing both ligands and equally impacting catalytic and thermodynamic efficiencies, respectively. In that light, residues associated with this helix could serve as molecular signatures for underlying drug-resistance mutations in *Pf*DHFR. Altogether, despite pairwise Pearson’s correlation of Δ*BC* (Figure S13) failing to group the different mutants based on respective degrees of resistance to pyrimethamine as discussed before [15] (WT: sensitive; SM: low resistant; DM1, DM2: moderately resistant; TM1, TM2: high resistant; and QM: highly resistant), these calculations provided useful clues related to catalytic efficiency. This is expected since DRN analysis highlights differences in intra-protein communication (which relates to protein function) when applied in comparative analysis between WT and mutant [22,37]. These findings further suggest that the pyrimethamine resistance mechanism is based on localized adjustments due to mutations within the *Pf*DHFR active site and may not correlate to its catalytic activity.

#### 2.3.3. Pyrimethamine Binding Confers Unique Residue Communication Changes Across Different Mutants

**Figure 9:**
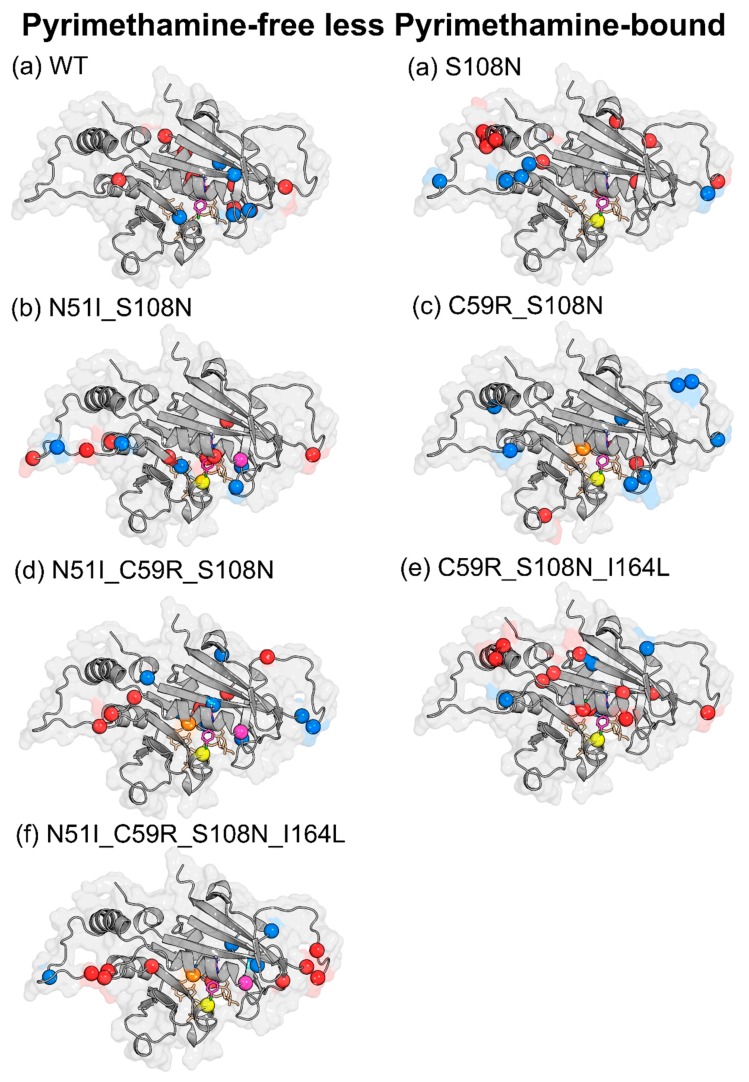
Effects of pyrimethamine binding on intra-protein communication: Structural mapping of residues showing significant changes in average *BC.* Changes in residue centrality (Δ*BC*) values were obtained by calculating pyrimethamine-free less pyrimethamine-bound average *BC* values. Residues with significant changes in *BC* were obtained by using a cutoff value of +/−2 SD of Δ*BC* values for each system. Red spheres represent residues with positive Δ*BC* values, while blue spheres are residues with negative Δ*BC* values. Also shown in spheres are pyrimethamine-resistant point mutations investigated in this study: Figure 8 coloring scheme was implemented here as well.

In order to gain insights into pyrimethamine-induced effects on protein communication, we calculated Δ*BC* values for each system as follows (pyrimethamine-free less pyrimethamine-bound) (Figure S14). Table 2 shows the residues with significant changes in average *BC*, obtained by applying a cutoff value of +/−2 SD of the BC changes for each mutant system. Figure 9 presents the residues with significant Δ*BC* values mapped to *Pf*DHFR structure. Positive Δ*BC* values denote a decrease in residue connectivity due to pyrimethamine binding, while negative Δ*BC* values denote increased importance of associated residues. Unlike mutation effects (Section 2.3.2) where there was a balance in the number of enhanced and diminished residue connectivity, an imbalance was observed following pyrimethamine binding. This was mainly seen as the number of residues with diminished connectivity doubled over the number with enhanced connectivity (Table 2, Figure 9, and Figure S14). Similar distant effects as noticed with mutations were also seen following pyrimethamine binding within the active site (Figure 9). Pairwise correlation of Δ*BC* values in this case revealed that each system responds to pyrimethamine binding in a rather unique manner as no two systems were seen to correlate with each other (r = −0.04–0.23) (Figure S14C,D). QM was shown to be the most unique compared to the rest of the mutants (r = 0.003–0.1), and its highest difference (r = 0.003) was with WT. This wide difference might relate to their sensitivity to pyrimethamine binding, given that WT is the most sensitive while QM is the most resistant mutant. Accordingly, detailed analysis of residue *BC* changes in WT and QM revealed that WT uniquely yielded enhanced *BC* values for Cys17, Leu46, Pro47, and Gly105, while Glu21, Gly41, Tyr158, Ser167, Glu175, Lys180, and Asp194 registered a decrease. On the other hand, QM yielded enhanced *BC* values for Thr36, Gly39, Asn90, and Leu164, while decreased values were registered in Ser22, Lys27, Ser95, Asn100, Lys155, and Tyr191. Despite a lack of functional information, it is likely that changes in residue *BC* involving these distinct residues might serve as molecular identifiers that ultimately determine associated levels for pyrimethamine sensitivity/resistance in *Pf*DHFR. 

## 3. Materials and Methods 

### 3.1. Homology Modeling

Homology modeling is an important computational technique that is useful in determining the 3D structure of proteins with unknown structural information by using available similar protein structures with high resolution as templates. Despite availability of 3D information for WT, DM1, and QM proteins in the Protein Data Bank (PDB), the structures possess missing residues (Val86–Ser95 (chain A) and Asn82–Lys96 (chain B)) in the DHFR domain and the junction region (Lys232–Asp282). For this reason, these proteins were subjected to homology modeling. Using MODELLER version 9.15 [38], the following structures were built and validated: WT, SM, DM1, DM2, TM1, TM2, and QM. Initially, the template structure PDB ID: 3QGT was identified as the best template using PRIMO web server [39]. Multiple sequence alignment was performed using PROMALS3D [40]. For mutated systems, mutations were manually introduced prior to modeling. One hundred models were calculated for each system, generating a total of 700 structures. Normalized Discrete Optimized Protein Energy (z-DOPE) score was used for global assessments and initial ranking of the generated models, and further quality evaluations were done using RAMPAGE [41], VERIFY3D [42], and ProSA [43] web servers. Briefly, the DOPE score is an atomic distance-dependent statistical potential derived from selected native structures which accounts for the finite and spherical shape of native structures and is normalized (Z-DOPE) by the number of all possible pairs of heavy atoms in the protein [38]. On the other hand, RAMPAGE provides ϕ and ψ angle plots, which encapsulate quite concise and intuitive protein backbone conformational information, while VERIFY3D provides a score of the compatibility of the modeled 3D structure with its amino acid sequence and, finally, ProSA performs a statistical comparison of the modeled structure against all available protein structures and highlights poorly modeled areas. The best model was chosen based on a consensus from these evaluations. Structure visualization and editing was done in Discovery studio visualizer version 4.1 [44] and PyMOL [45].

### 3.2. Molecular Docking

Prior to the docking experiment, the 3D structure of pyrimethamine was obtained from PubChem web server [46]. Pubchem is a chemical information resource at the U.S. National Center for Biotechnology Information (NCBI) and contains millions of unique chemical structures. Protein and ligand preparations were done using AutoDock4 tools [47]. Briefly, ligand preparation was done using prepare_ligand4.py, while proteins were prepared using prepare_receptor4.py tools. Molecular docking simulations were performed using AutoDock Vina [48]. A blind docking protocol was implemented whereby the entire surface of DHFR was screened. Initially, docking validation was achieved by redocking pyrimethamine into the active site of the modeled WT *Pf*DHFR structure and by comparing its binding orientation and interactions to that of the WT crystal structure (PDB ID: 3QGT). Parameters used here were adopted for all docking experiments. These included a grid box search space of 60 Å × 60 Å × 60 Å; box center of x = 26.17, y = −32.25, and z = 13.91; and an exhaustiveness search value of 360. Each docking experiment generated ten pyrimethamine–DHFR complexes which were ranked using Vina scores. Complexes yielding the lowest scores were considered for proceeding experiments (MD simulations). Protein–ligand interactions were visualized, and figures were generated using LIGPLOT+ [49].

### 3.3. Molecular Dynamics

Molecular dynamics simulations were performed on the wild type pyrimethamine complex (WT-PYR), the six mutant pyrimethamine complexes (MTs-PYR), and the pyrimethamine-free wild type. All systems were complexed with endogenous NADPH. Simulations were performed using GROMACS v.2016 package [50] at the Center for High Performance Computing, Cape town. Systems were embedded in explicit TIP3P water molecules and enclosed by a cubic simulation box with at least 2 Å buffer from the edge of the protein. AMBER03 forcefield [51] parameters were employed for topology generation. Ligand parameters were determined using the ACPYPE tool [52]. Total system charges were neutralized using 0.15 M Na^+^ and Cl^−^ counterions. The systems were energy minimized using the steepest descent algorithm up to a maximum force threshold value of ≤1000 kJ/mol/nm. Short-range non-bonded contacts were defined at a 1.4-nm cutoff while long-range interactions were treated using the Particle Mesh-Ewald (PME) method. Coupled with time constants of 0.1 ps, systems were temperature equilibrated at 300 K to a modified Berendsen thermostat, succeeded by pressure equilibration at 2-ps time constant and a reference 1 bar pressure to Parrinello−Rahman barostat; 100,000 steps were applied in each case. Covalent bonds were constrained using Lincs algorithm. With periodic boundary conditions set, 100-ns production runs at 2-fs timesteps were performed.

#### 3.3.1. Trajectory Analysis

Initially, trajectories were corrected for periodic boundary conditions using *trjconv* gromacs tool. Besides total pressure and temperature, systemic energies, including potential and kinetic, were checked to assess simulation quality. The root mean square deviation (RMSD), root mean square fluctuation (RMSF), radius of gyration (Rg), and hydrogen bond numbers were calculated using standard Gromacs tools. To analyze prolonged protein–ligand interactions, ligand RMSDs of the last 15 ns were clustered using the *gmx cluster* tool while implementing geometric clustering method described by Daura et al. [53].

#### 3.3.2. Thermodynamic Assessment

Through the *g_mmpbsa* module [54], binding free energies between proteins (wild type and mutants) and pyrimethamine were computed using the Molecular Mechanics Poisson−Boltzmann Surface Area (MM-PBSA) method [26] on trajectory snapshots spanning equilibrated 15-ns periods indicated in Table S7. Frames were sampled at 50 ps time intervals. In total, calculations were performed on 750 frames. Fundamentally, bound (complex) and unbound (receptor and ligand) end states are considered in these calculations. Binding free energy between protein–ligand complexes can be estimated using the following equations:

(1) $$\Delta G_{bind}=\Delta G_{complex}-\left(\Delta G_{receptor}-\Delta G_{ligand}\right)$$

(2) $$\Delta G_{x}=Emm-\left(TS+G_{solv}\right)$$

where $\Delta G_{complex}$, $\Delta G_{receptor}$, and $\Delta G_{ligand}$ represent free energy values of the complex, receptor, and ligand, respectively. The Gibbs free energy ($\Delta G_{x}$) for each component is determined by calculations of solvation free energy ($G_{solv}$), molecular mechanics energy (Emm), and entropic potential (T:temperature, S:Entropy). In practice, $\Delta G_{x}$ is computed from estimates of Emm (van der Waals and electrostatic interactions) and $G_{solv}$ (polar and nonpolar solvation free energy) energy terms using the *g_mmpbsa* tool [54]. To quantify the contribution of specific residues to binding, free energy was decomposed on per residue basis.

#### 3.3.3. Essential Dynamics

To comprehensively assess the effects of mutations on conformational redistributions, principal component analysis (PCA) was performed on trajectories of the pyrimethamine-free and pyrimethamine-bound systems. Based on positional fluctuation of protein backbone atoms, a covariance matrix was built using the *gmx_covar* tool. Considering a trajectory of *m* observations associated with a protein of *N* backbone atoms, the covariance (*C*) is a 3*N*X*N* matrix in which an element $C_{ij}$ is given by the following:

(3) $$C_{ij}=\left(X_{i}-{\bar{X}}_{i}\right)\left(X_{j}-{\bar{X}}_{j}\right)$$

where *i* and *j* specify each of the 3*N* Cartesian coordinates. ${\bar{X}}_{i}$ and ${\bar{X}}_{j}$ represent the respective ensemble average accrued from *m* observations. Eigenvalue decomposition of *C* was performed using the *gmx_anaeg* tool to obtain eigenvectors ordered based on descending eigenvalue indices. Often, the first two eigenvectors (PC1 and PC2) explain data with the highest variance and hence functionally relevant motions. To illustrate the differences in protein folding or unfolding events during simulation, free-energy landscapes (FEL) were constructed in the space of the top two PC’s (PC1 and PC2) using the *gmx_sham* tool.

#### 3.3.4. Dynamic Residue Network Analysis

Dynamic residue network (DRN) analysis was performed on trajectories to identify residues important for communication in the WT as well as to reveal local changes in residue interaction network signatures because of initiated mutations and pyrimethamine binding. Two fundamental network properties were computed: average *L* and average *BC*. We describe the system in a set of nodes and edges, where an edge (link) between two nodes (residues) was defined to have formed if the Cβ (Cα for Glycine) atoms approach a distance of ≤6.7 Å. An existing link is assigned a scalar 1, while no link equals 0. Residue interaction network (RIN) graphs were constructed using the NetworkX package of the MD-TASK tool [37]. Considering a protein having total residues *j*, the average shortest path index of a residue *i* can be determined by calculating the mean pairwise distances (*D*) to each residue within the protein network. Each one of the pairwise distances (*D_ij_*) describes the path bearing the least number of links from all possible paths between *i* and *j*. Average shortest path (*L*) denotes how far down a residue is in terms of reachability from all other residues for communication. *BC* index highlights how frequent a residue participates in the shortest paths between all residue pairs. DRN reports the moving average of RIN graphs and hence average *L* and average *BC*. In this case, RIN graphs were aggregated over the last 15 ns periods (Table S7) at timestep intervals of 10 ps.

## 4. Conclusions

To date, numerous computational approaches have been applied towards understanding drug-resistance-related mechanisms. Here, we utilized molecular modeling and dynamic residue network (DRN) analysis concepts to elucidate mechanisms of action of pyrimethamine-resistant mutations in *Pf*DHFR. Assessment of global effects of mutations on *Pf*DHFR revealed subtle but considerable impacts of mutations on conformation selection. Generally, mutations destabilized *Pf*DHFR, generating more mobile (flexible) systems. Specifically, DM1 (N51_S108N), DM2 (C59R_S108N), and TM2 (C59R_S108N_I164L) enhanced conformational plasticity while TM1 favored conformational rigidity in both pyrimethamine-free and pyrimethamine-bound systems. Since mutations were located within the ligand binding site, ligand RMSDs were computed to assess their impact on pyrimethamine’s binding pose. Interestingly, pyrimethamine occupied multiple conformations across all systems during simulation. For the WT, two dominant conformations were adopted as a result of conformational isomerism of the 4-chlorophenyl group of pyrimethamine. The QM (N51I+C59R+S108N+I164L) displayed the largest conformation heterogeneity depicting a weakly bound ligand. Also, binding free-energy calculations generally depicted diminished protein–ligand binding affinity with increased number of mutations. These findings agreed with previous in vivo and in vitro studies proposing QM as the most resistant form. DRN analysis identified highly connected residues, including Asp10, Tyr12, Ile14, Cys15, Ala16, Met55, Lys181, and Thr185, previously implicated in substrate and cofactor interactions. Residues Cys17, Gly41, and Pro198 based on residue accessibility (average *L*) results and residues Ser167, Val168, Tyr170, and Gln171 based on residue connectivity (average *BC*) were conspicuously identified in all mutants. It is likely that the above residues formulate useful molecular fingerprints that ultimately determine the functional separation between WT and mutated systems. It was evident that the WT system is extremely different from QM following changes in average BC due to pyrimethamine binding. Among the four pyrimethamine-resistant point mutations in *Pf*DHFR, Cys59 was identified as the only residue with high average *BC* value in the WT. Mutations involving Cys59 (DM2, TM1, TM2, and QM) have demonstrated poor catalytic properties in in vivo studies. This suggests that drug-resistance mutations targeting residues with high connectivity could have a detrimental effect on parasite fitness. Although DRN analysis failed to group the different mutants based on respective degrees of resistance to pyrimethamine (sensitive WT, low resistance SM, moderately resistant DM1, DM2, high resistance TM1 and TM2, and highly resistant QM), it provided useful clues relating to their catalytic efficiency. These findings suggest that the pyrimethamine-resistance mechanism is based on localized changes due to mutations within the *Pf*DHFR active site and may not be related to its catalytic activity.

## Figures and Tables

**Table 1:** Thermodynamic analysis: Comparisons of binding energy between wild type and mutated DHFR–pyrimethamine complexes. Energy values in parenthesis represent the sd (standard deviation). Key: van der Waals: Δ*E*_vdW_, Electrostatic: Δ*E*_elec,_ polar solvation: ΔG_polar_, nonpolar: ΔG_nonpolar_.

Systems	Δ*E_vdW_*	Δ*E_elec_*	Δ*G_polar_*	Δ*G_nonpolar_*	Δ*G _binding_* (kJ mol^−1^)
*WT*	−145.40 (0.20)	−5.97 (0.06)	38.76 (0.10)	−14.58 (0.02)	−127.20 (0.21)
*S108N*	−136.80 (0.19)	−7.32 (0.06)	50.06 (0.15)	−14.21 (0.02)	−108.26 (0.21)
*N51I_S108N*	−124.09 (0.19)	−6.63 (0.06)	32.05 (0.11)	−14.46 (0.02)	−113.12 (0.20)
*C59R_S108N*	−127.74 (0.21)	−5.28 (0.07)	62.50 (0.16)	−13.90 (0.02)	−84.40 (0.23)
*N51I_ C59R_S108N*	−130.53 (0.24)	−4.29 (0.06)	47.76 (0.18)	−13.82 (0.02)	−100.87 (0.26)
*C59R_S108N_I164L*	−105.48 (0.26)	−2.59 (0.06)	28.69 (0.11)	−13.40 (0.02)	−92.77 (0.25)
*N51I_C59R_S108N_I164L*	−116.49 (0.18)	−6.98 (0.08)	53.07 (0.21)	−14.49 (0.02)	−84.90 (0.25)

**Table 2:** Changes in *betweenness centrality (BC)*: Table of residues showing significant changes in average *BC* (Δ*BC*) due to mutation (pyrimethamine-free: WT-free less mutant-free; pyrimethamine-bound: WT-PYR less mutant-PYR) and pyrimethamine binding (PYR-free less PYR-bound). In bold are residues showing changes in *BC* at sites with high centrality, while ↑ and ↓ signify residues with significant positive and negative Δ*BC* values, respectively, obtained by using a threshold value of +/−2 SD of the Δ*BC* values for each system. Positive Δ*BC* signify decrease in residue connectivity while negative Δ*BC* stands for increased residue connectivity and hence increased residue participation in communication.

Effect of Mutation (pyrimethamine-free: WT-free less mutant-free)		
Protein	Δ*BC*	Residues
*S108N*	↑	**Ala13, Ala16, Cys18**, Gly41, **Tyr158**, Tyr159, Gly165, Ser167, Thr185
	↓	**Ile14, Tyr35**, Thr36, Ile143, **Lys160**, **Val168**, **Gln171**, **Pro198**, **Asn201**
*N51I_S108N*	↑	Gly41, Gly165, Ser167, **Thr185**
	↓	Lys23, Asp91, **Asn157**, **Gln171**, **Pro198**
*C59R_S108N*	↑	Lys49, Cys78, Lys79, Gly165, Thr185, Phe196
	↓	Thr36, Asn51, Asp81, **Arg106**, Lys132, **Asn157**, Val168, **Gln171**, Ile200
*N51I_C59R_S108N*	↑	**Ala16, Cys18**, Glu21, Gly41, **Val103**, Gly165, Ser167
	↓	Phe32, Asn33, Tyr35, Lys96, Val168, **Gln171, Pro198**
*C59R_S108N_I164L*	↑	**Ala13**, **Cys15**, **Gly41**, Tyr158, Gly166, Thr185, Phe196
	↓	Ser22, **Glu71**, Lys72, **Lys160**, **Gln171**, **Lys181**, **Pro198**
*N51I_C59R_S108N_I164L*	↑	Ile11, Gly41, Lys79, Tyr158, Gly165, **Ser167**, **Thr185**, Phe196
	↓	Ser22, Ser95, Ile112, Asn124, Val168, **Gln171**, **Pro198**
**Effect of Mutation (pyrimethamine-bound: WT-PYR less mutant-PYR)**		
*S108N*	↑	**Cys17**, **Gly26**, **Leu46**, **Cys78**, **Gly105**, **Trp109**, **Gly165**, **Gly166**
	↓	Lys97, **Val101**, Ser167, Val168, **Tyr170**, **Gln171**
*N51I_S108N*	↑	Cys17, Leu46, Pro47, Met55, **Gly105**, Gly166, Leu174
	↓	**Glu21**, **Gly41**, **Tyr158**, **Tyr159**, **Ser167**, Tyr170, **Gln171**
*C59R_S108N*	↑	Ile11, Cys17, Val20, Leu46, Pro47, **Gly105**, Trp109, Gly165
	↓	Asn24, Phe32, **Thr36**, Lys97, Ser167, Val168, **Tyr170**, **Gln171**
*N51I_C59R_S108N*	↑	Cys17, Leu46, Pro47, **Gly105**, Trp109
	↓	**Cys15**, Tyr35, **Gly41**, Ser167, **Val168**, **Lys180**, **Asn201**
*C59R_S108N_I164L*	↑	**Ala16**, Cys17, Leu46, Pro47, Met55, Arg59, **Gly105**, Trp109, Gly165, Lys181
	↓	Ser22, **Ser167**, **Val168**, **Tyr170**, **Gln171**, **Pro198**, Thr220
*N51I_C59R_S108N_I164L*	↑	Cys17, Val20, Leu46, Pro47, **Gly105**, Trp109, Gly165
	↓	**Glu21**, Gly41, Asn90, **Ser167**, Val168, **Tyr170**, **Gln171**, **Lys180**, **Pro198**
**Effect of Pyrimethamine (PYR-free less PYR-bound)**		
*WT*	↑	Glu21, Gly41, Tyr158, Ser167, Tyr170, Glu175, **Lys180**, Asp194, Phe196
	↓	**Cys17**, **Leu46**, **Pro47**, **Gly105**, **Pro198**
*S108N*	↑	Asn24, Tyr35, Glu71, Cys78, Lys79, Lys160, Val168, Phe196, AS201
	↓	Lys23, Val89, Asn157, Tyr158, **Tyr159**, **Gln171**
*N51I_S108N*	↑	Lys23, Met55, Asp91, Ser95, **Val103**, Asn157, Gly166, **Pro198**
	↓	**Gly41**, **Val45**, **Pro93**, **Leu98**, **Met104**, **Ser167**
*C59R_S108N*	↑	**Gly41**, Asp81, Met81, **Lys132**, **Ser167**
	↓	Asn24, Val31, Phe32, Val45, Lys49, Ser81, Lys81, Lys97, **Gln171**, **Pro198**
*N51I_C59R_S108N*	↑	Asn33, Lys96, Asn157, Tyr159, Tyr170, **Gln171**, Pro198
	↓	**Asp10**, **Cys15**, Ser22, Lys23, Gly41, **Ile163**, **Val168**, **Asn201**
*C59R_S108N_I164L*	↑	Ile11, **Ala16**, **Cys18**, Lys23, Ser52, Arg59, Glu71, Lys72, Lys160, Gly165, Lys181
	↓	Asn34, Asn157, **Ile163**, **Val168**, **Gln171**, Ile182
*N51I_C59R_S108N_I164L*	↑	Ser22, Lys23, Lys27, Ser95, Lys96, Asn100, Lys155, Gly166, Tyr191
	↓	Thr36, **Gly39**, Asn90, **Leu164**, **Pro198**

## Supplementary Materials

The following are available online. Table S1: Validation of homology modeled structures, Table S2: Docking scores of pyrimethamine in wild type and mutants, Figure S1: Molecular docking poses and interactions visualized using LigPlot+, Figure S2: RMSD evolution of protein backbone atoms during 100-ns simulation, Table S3: Calculated average protein RMSD values, Figure S3: Ligand RMSDs, Figure S4: Radius of gyration (*Rg*) plots depicting structural compactness over time, Figure S5: Histograms of the radius of gyration (*Rg*). Table S4: Principal component analysis, Figure S6: Principal component analysis results of both WT and mutated pyrimethamine-bound and pyrimethamine-free *Pf*DHFR, Table S5: Computed trace values (sum of 2079 eigenvalues) of diagonalized covariance matrices for each model, Figure S7: Total binding free energy decomposed on per residue basis, Table S6: Molecular Mechanics Poisson−Boltzmann Surface Area (MM-PBSA) analysis, Figure S8: Hydrogen bond numbers yielded during 100-ns simulation, Figure S9: DHFR structure with mapped communication hubs (high *BC* centres), Figure S10. Effect of mutation on residue centrality (WT-free less mutant-free), Figure S11. Effect of mutation on residue centrality (WT-bound less mutant-bound), Figure S12: Structural mapping of residues that yielded large changes in average *BC* values (WT-PYR less mutant-PYR) for pyrimethamine-bound *Pf*DHFR models, Figure S13: Pairwise Pearson’s correlation heatmap of average BC differences (ΔBC), Figure S14: Effects of ligand binding on residue centrality (pyrimethamine-free less pyrimethamine-bound), Table S7: Summary of equilibrated trajectory regions sampled for analyses of binding free energy and dynamic residue interaction network (DRN).
