# Supplementary material for: Understanding the Pyrimethamine Drug Resistance Mechanism via Combined Molecular Dynamics and Dynamic Residue Network Analysis
Source: Molecules. 2020 Feb 18;25(4):904. doi: 10.3390/molecules25040904 (PMC7070769; doi:10.3390/molecules25040904)
Supplement: Supplementary file 1 [file molecules-25-00904-s001.zip › molecules-676872-SI/Supplementary_Figures/Amusengeri_etal_SD_December2019_Molecules.pdf]

Supplementary Material

Understanding the pyrimethamine drug resistance mechanism via combined molecular dynamics and dynamic residue network analysis

*Arnold Amusengeri[1a, †], Rolland Bantar Tata[1b, †], and Özlem Tastan Bishop [1c,]**

[1]Research Unit in Bioinformatics (RUBi), Department of Biochemistry and Microbiology, Rhodes University, Grahamstown, 6140, South Africa; g16a7782@campus.ru.ac.za [1a], g18t9691@campus.ru.ac.za [1b], O.TastanBishop@ru.ac.za [1c]

Correspondence: O.TastanBishop@ru.ac.za; Tel.: +27-46-603-8072

† These authors contributed equally to this work

Table S1: Validation of homology modelled structures: Tabulated summary of Z-DOPE scores, Ramachandran values, Verify-3D percentages and ProSA z-scores.

Protein Model	Z-DOPE score	Ramachandran Plot			Verify3D (%)	PROSA (Z-Score)
		Number of favoured region residues (Percentage)	Number of allowed region residues (Percentage)	Number of outlier region residues (Percentage)		
Wild_type	-1.04	1072 (95.0)	47 (4.2)	9 (0.8)	86.4	-10.81
S108N	-1.07	1074 (95.2)	45 (4.0)	9 (0.8)	88.16	-10.5
N51I_S108N	-1.04	1079 (95.7)	44 (3.9)	5 (0.4)	85.6	-10.54
C59R_S108N	-1.05	1083 (96.0)	40 (3.5)	5 (0.4)	89.13	-10.57
N51I_C59R_S108N	-1.04	1078 (95.6)	44 (3.9)	6 (0.5)	87.28	-10.65
C59R_S108N_I164L	-1.04	1083 (96.0)	39 (3.5)	6 (0.5)	83.22	-10.8
N51I_C59R_S108N_I164L	-1.05	1082 (95.9)	39 (3.5)	7 (0.6)	83.13	-10.59

Table S2: Docking scores of pyrimethamine in wild type and mutants: Table showing Vina docking scores and DSX rescoring values.

**Protein**	**Pyrimethamine Vina Score (Kcal/mol)**	**RMSD**
wild_type	-9.4	0.658
S108N	-9.8	0.744
N51I_S108N	-8.4	5.904
C59R_S108N	-8.8	0.580
N51I_C59R_S108N	-8.4	0.849
C59R_S108N_I164L	-7.7	6.595
N51I_C59R_S108N_I164L	-9	1.033

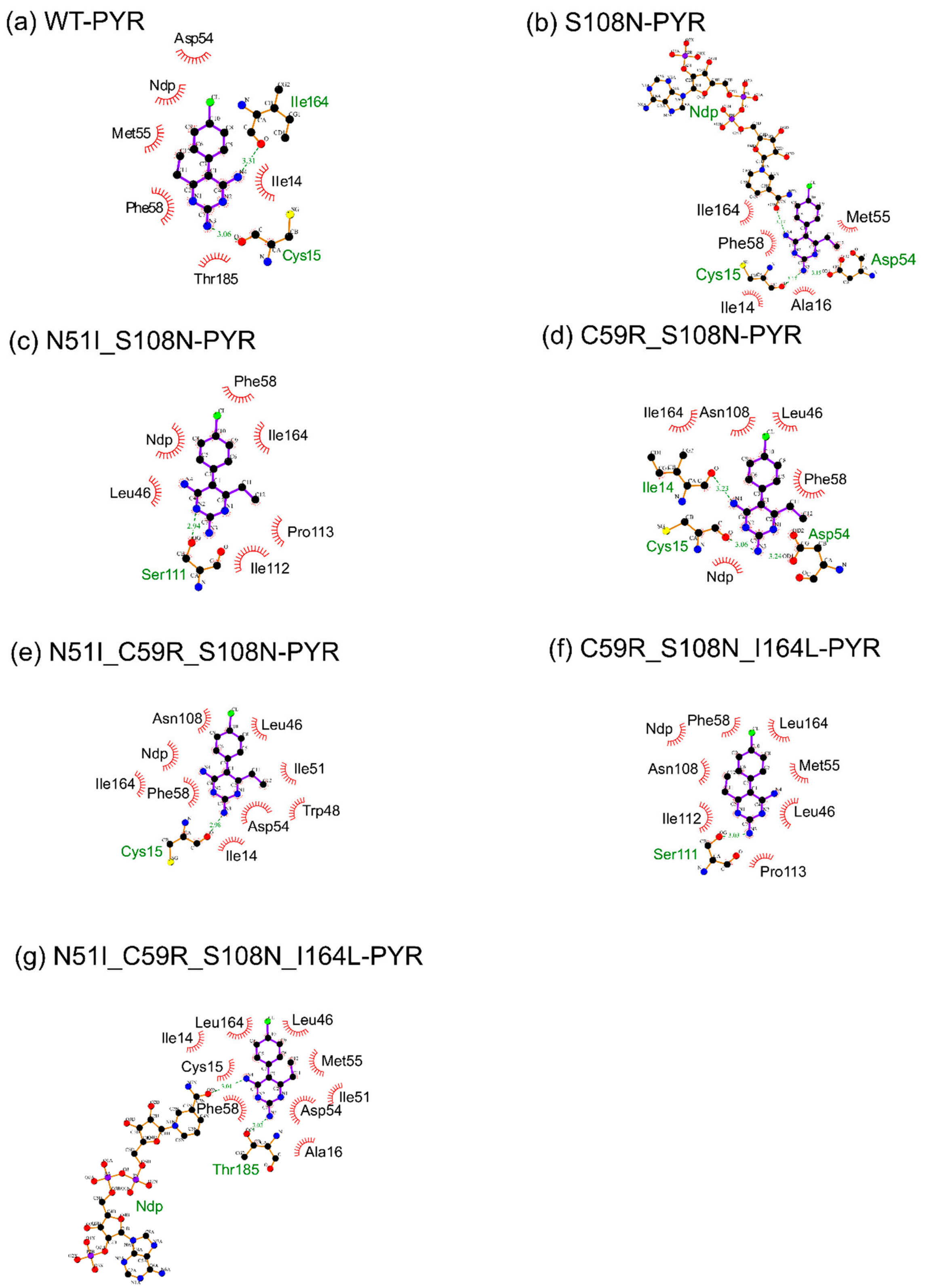

Figure S1: Molecular docking poses and interactions visualized using LigPlot+. (A) Shows binding pocket as transparent grey surface with pyrimethamine (purple) and NADP inside. Residues found around active site are shown as sticks. (B) Pyrimethamine is represented in ball and stick, with bonds shown as purple solid lines. Protein residues or NADPH forming hydrogen bonds are also represented in ball and stick with bonds shown as brown solid lines. Hydrogen bonds are shown as dashed green lines.

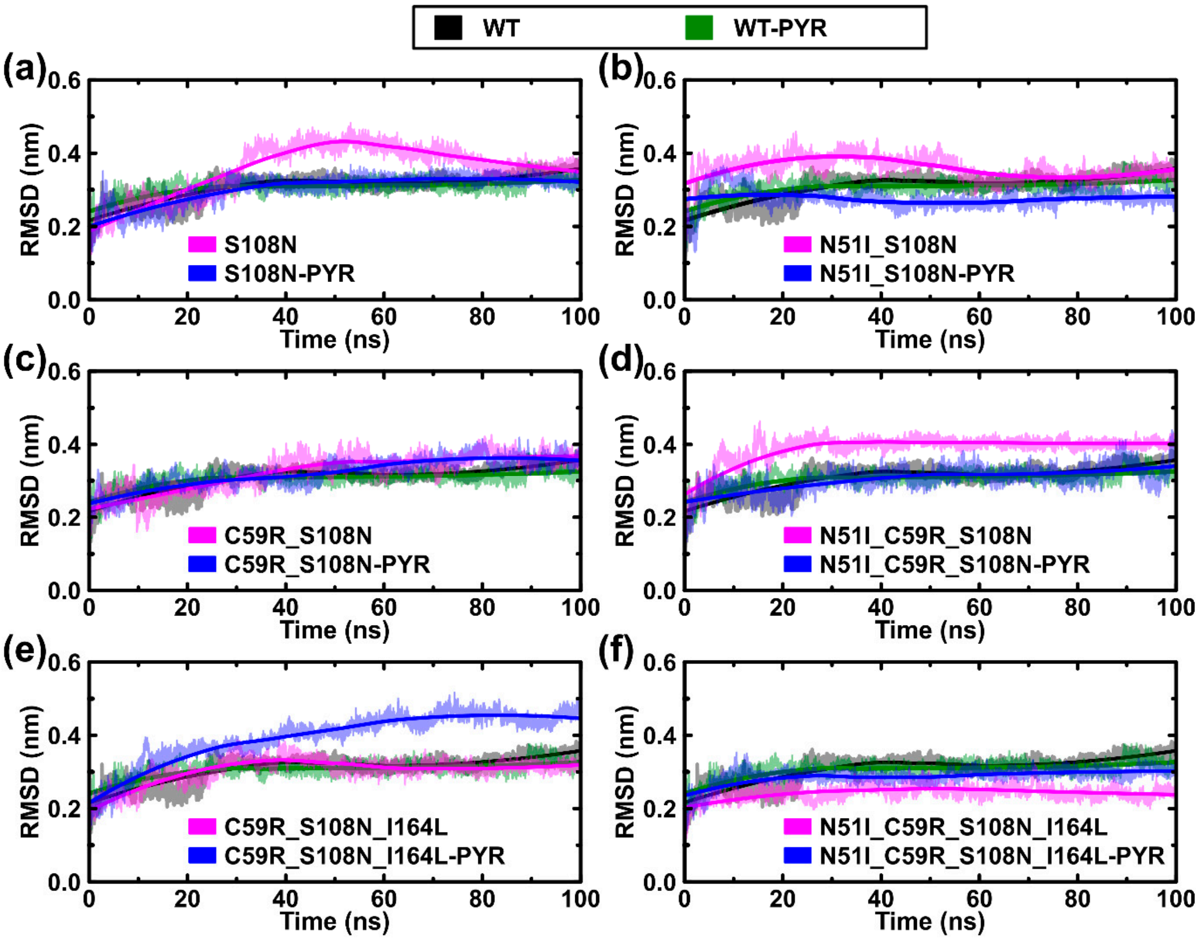

Figure S2: RMSD evolution of protein backbone atoms during 100ns simulation.

Table S3: Calculated average protein RMSD values.

	System	Average RMSD (nm)
Pyrimethamine-bound	Wildtype – PYR	0.31
	S108N – PYR	0.30
	N51I_S108N – PYR	0.28
	C59R_S108N – PYR	0.32
	N51I_ C59R_S108N – PYR	0.30
	C59R_S108N_I164L – PYR	0.40
	N51I_ C59R_S108N_I164L – PYR	0.29
Pyrimethamine-free	Wildtype	0.31
	S108N	0.36
	N51I_S108N	0.36
	C59R_S108N	0.32
	N51I_ C59R_S108N	0.39
	C59R_S108N_I164L	0.30
	N51I_ C59R_S108N_I164L	0.24

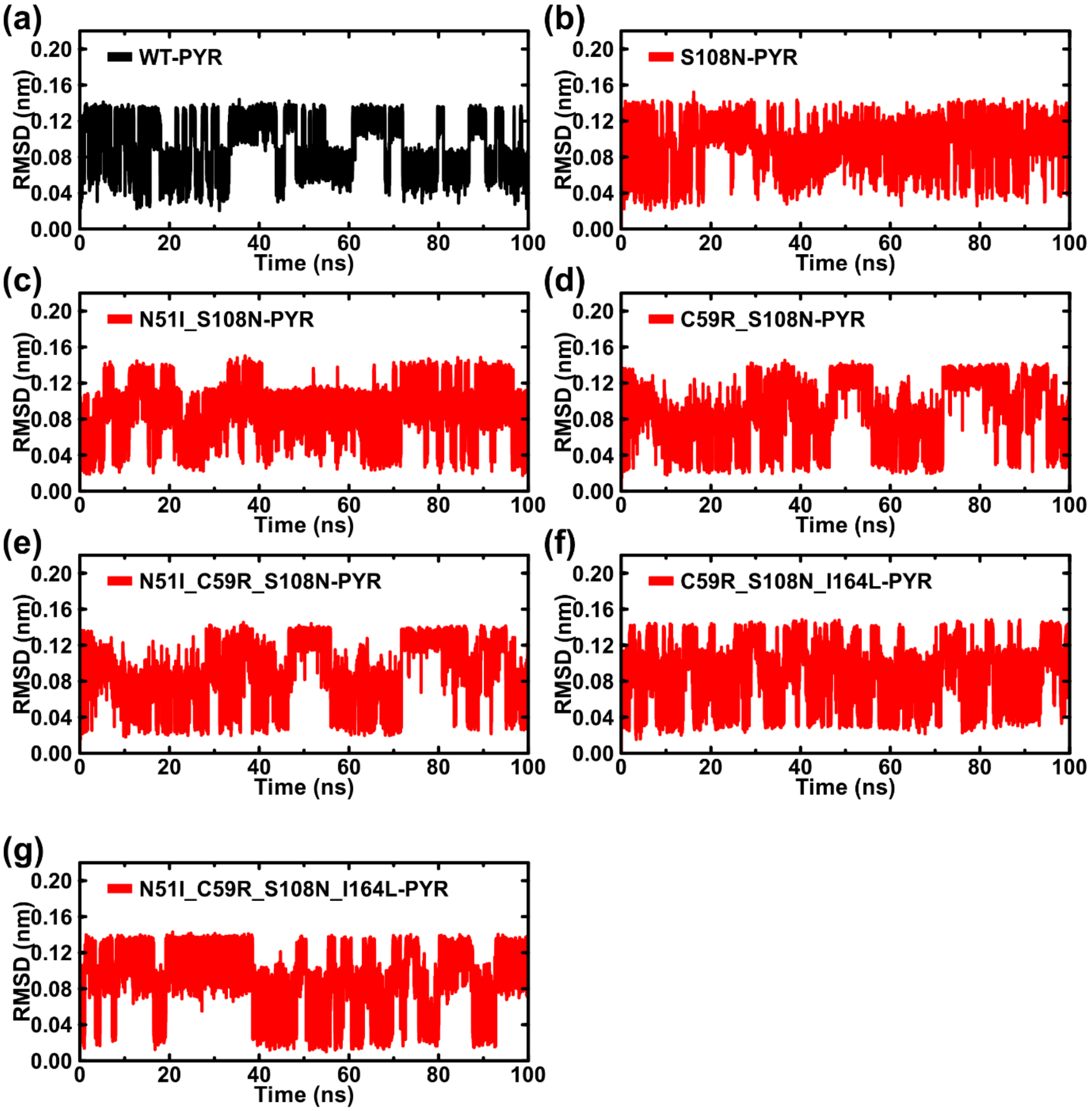

Figure S3: Ligand RMSDs: RMSD evolution of pyrimethamine complexed with WT and mutated PfDHFR during 100ns simulation. Color key: black: pyrimethamine-bound WT, red: pyrimethamine-bound mutants.

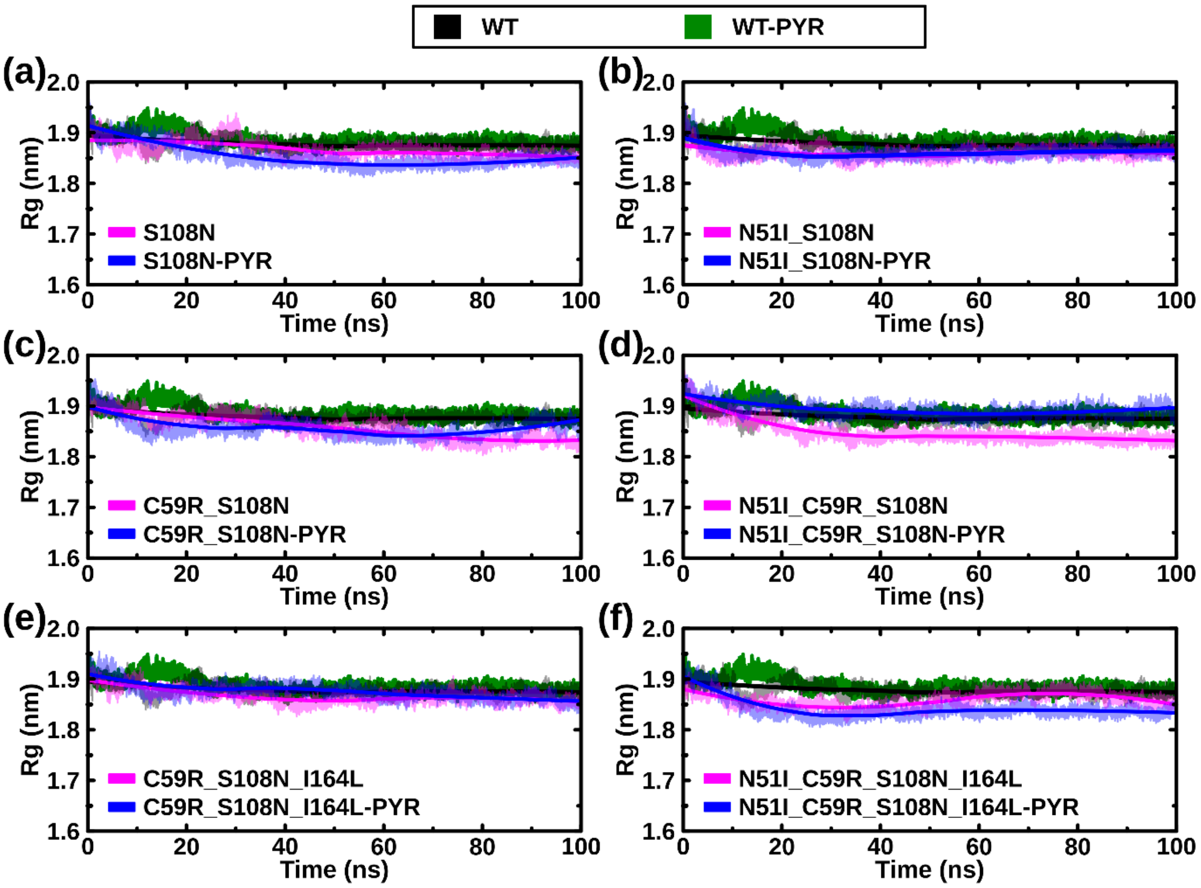

Figure S4: Radius of gyration (Rg) plots depicting the evolution of *pf*DHFR structure compactness over 100ns period.

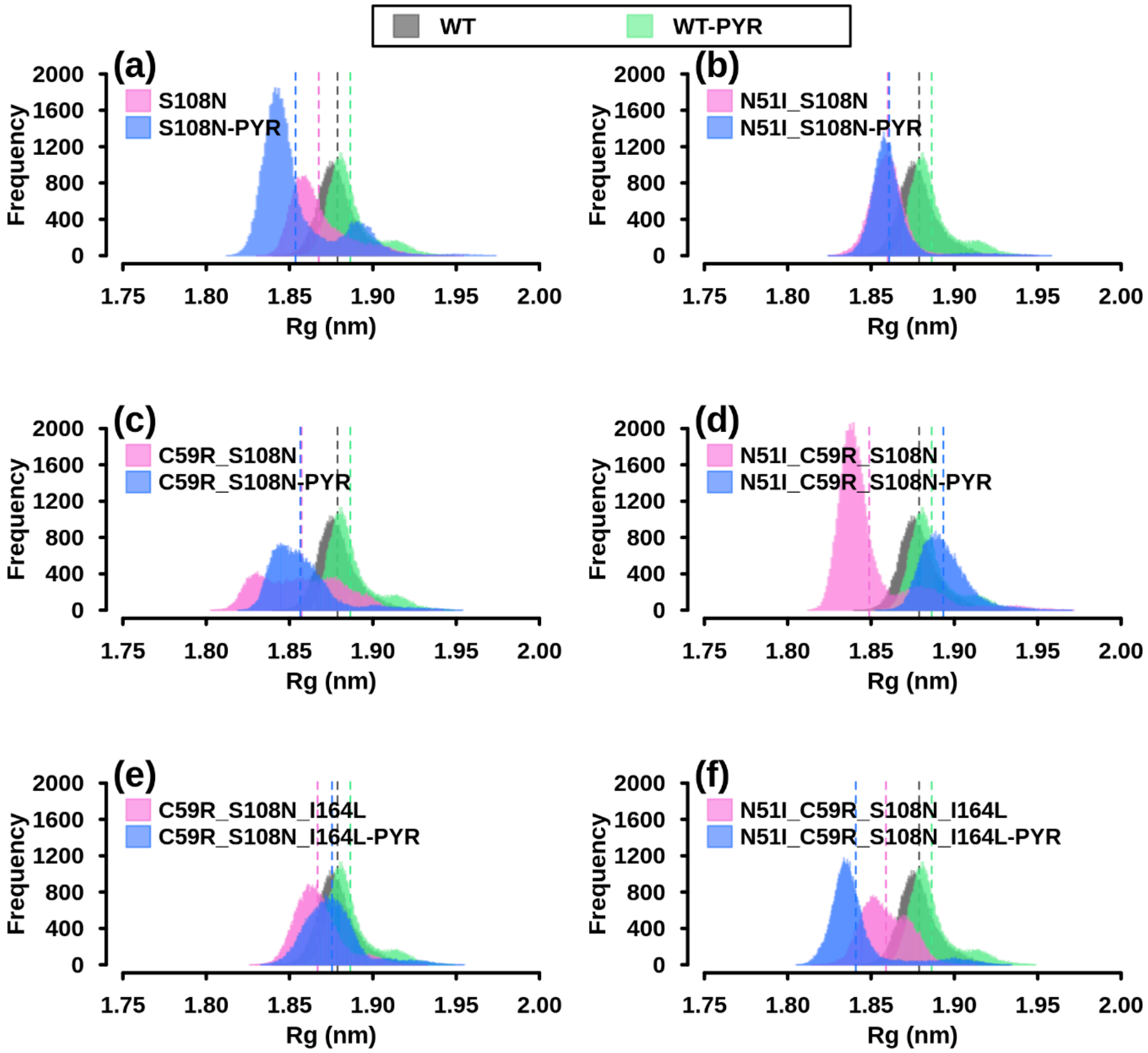

Figure S5: Histograms of the radius of gyration (*Rg*).

Table S4: Principal component analysis: Percentage variance contribution of the top two eigenvectors to overall motion.

System	Pyrimethamine free percentage variance		Pyrimethamine bound percentage variance	
	PC1	PC2	PC1	PC2
WT	23.95%	14.04%	46.15%	7.29%
S108N	46.84%	13.84%	35.21%	16.30%
N51I_S108N	34.48%	19.11%	21.91%	11.15%
C59R_S108N	34.09%	16.79%	31.45%	12.08%
N51I_ C59R_S108N	23.99%	18.27%	36.48%	13.16%
C59R_S108N_I164L	28.43%	13.54%	42.48%	16.62%
N51I_C59R_S108N_I164L	25.98%	15.51%	34.27%	10.37%

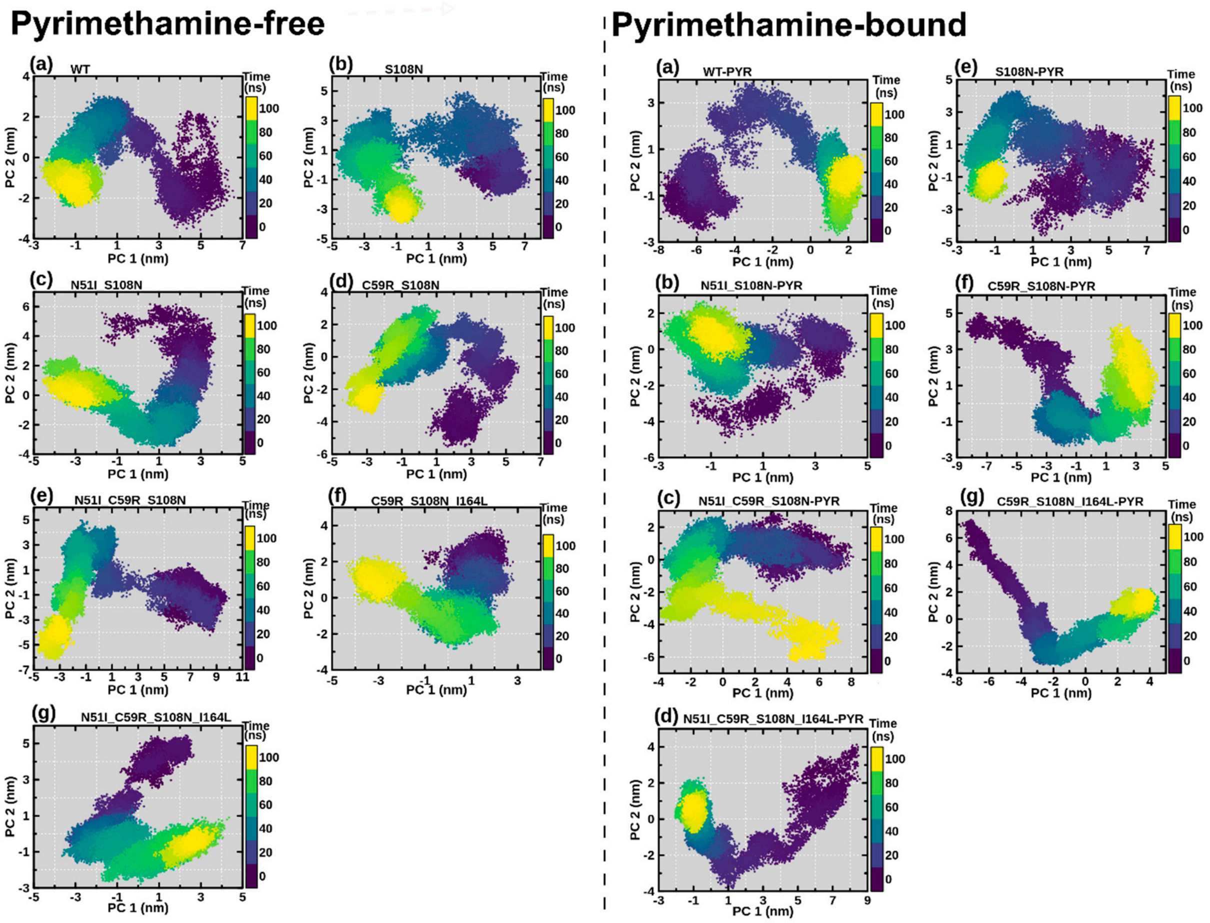

Figure S6: Principal component analysis results of both WT and mutated Pyrimethamine-bound and Pyrimethamine-free PfDHFR. 2D projections of the top two eigenvectors versus time evolution from black (0 ns) to yellow (100 ns).

Table S5: Computed trace values (sum of 2079 eigenvalues) of diagonalized covariance matrices for each model.

System	Pyrimethamine free	Pyrimethamine bound
	Trace value	Trace value
WT	13.0925	15.8886
S108N	23.3786	15.5614
N51I_S108N	14.0945	9.96848
C59R_S108N	22.6307	17.6195
N51I_ C59R_S108N	15.5534	18.3309
C59R_S108N_I164L	15.0566	22.2634
N51I_C59R_S108N_I164L	13.6105	11.6472

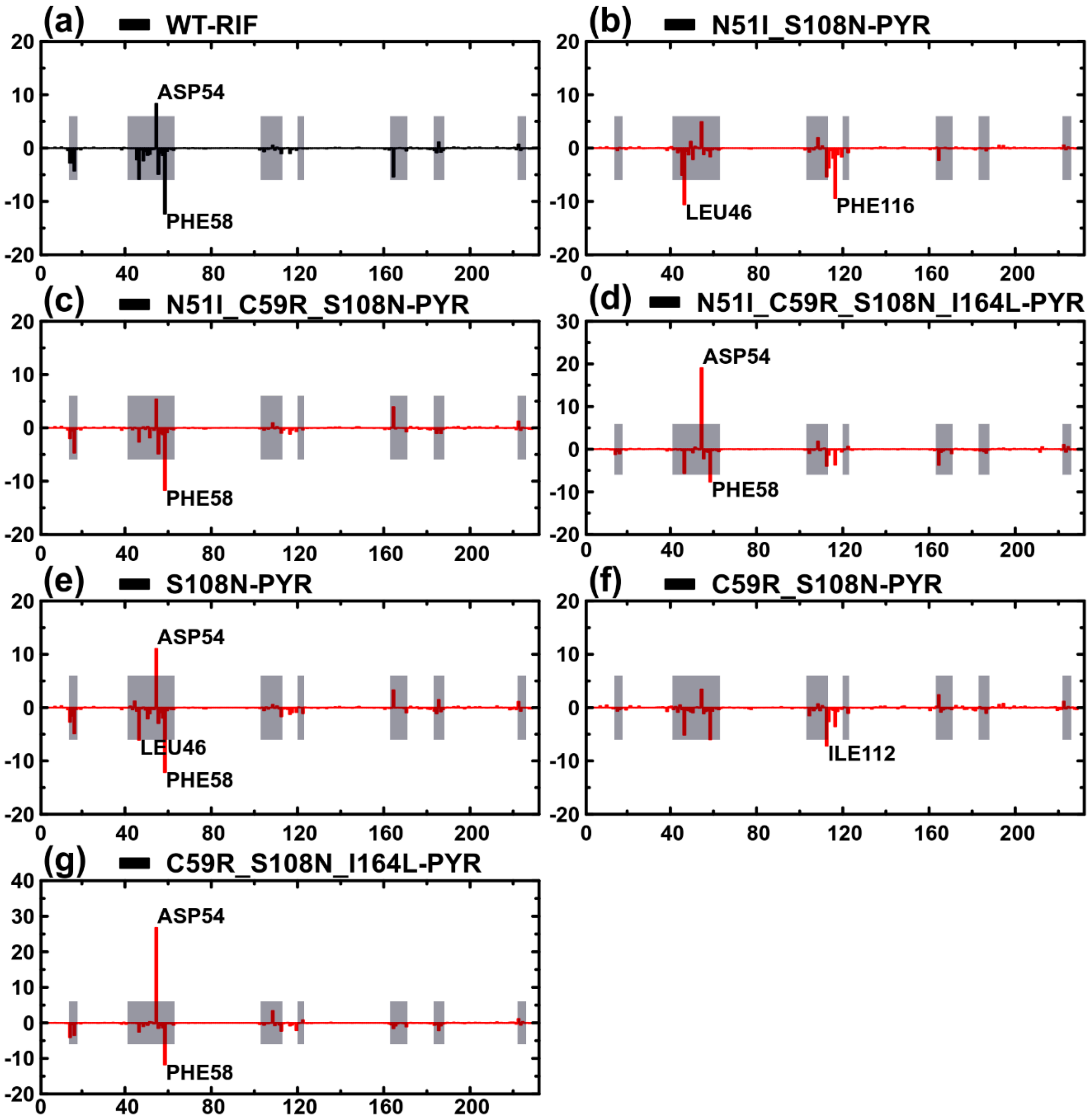

Figure S7: Total binding free energy decomposed on per residue basis. Residues that yielded significant contribution to binding free energy were labelled.

Table S6: MMPBSA analysis. Summary of residues that yielded substantial binding free energy changes (more than 2 kJ/mol) because of mutation (s).

System	Energy change (wildtype less mutated)			
	Residue number	Energy difference: -ve (kJ/mol)	Residue number	Energy difference: +ve (kJ/mol)
S108N	164<br>54<br>48<br>55	-8.62<br>-2.71<br>-2.41<br>-1.97	-	-
N51I_S108N	58<br>16<br>55<br>164<br>14<br>15	-10.74<br>-4.35<br>-3.73<br>-3.08<br>-2.69<br>-2.32	45<br>54<br>113<br>112<br>46<br>116	2.90<br>3.42<br>3.59<br>4.33<br>4.75<br>8.42
C59R_S108N	164<br>58<br>16<br>55<br>14<br>15	-7.71<br>-6.35<br>-3.89<br>-3.80<br>-2.65<br>-2.10	113<br>116<br>54<br>112	2.47<br>2.55<br>4.91<br>6.16
N51I_ C59R_S108N	164<br>46<br>48<br>15<br>45	-9.26<br>-3.21<br>-2.49<br>-2.19<br>-2.01	54	2.98
C59R_S108N_I164L	54<br>164<br>55<br>46<br>108<br>15<br>45	-18.42<br>-3.82<br>-3.42<br>-3.29<br>-2.87<br>-2.31<br>-2.01	185	3.22
N51I_C59R_S108N_I164L	54<br>58<br>16<br>55<br>15<br>48	-10.71<br>-4.72<br>-3.22<br>-2.68<br>-2.29<br>-2.15	116<br>112	2.70<br>2.92

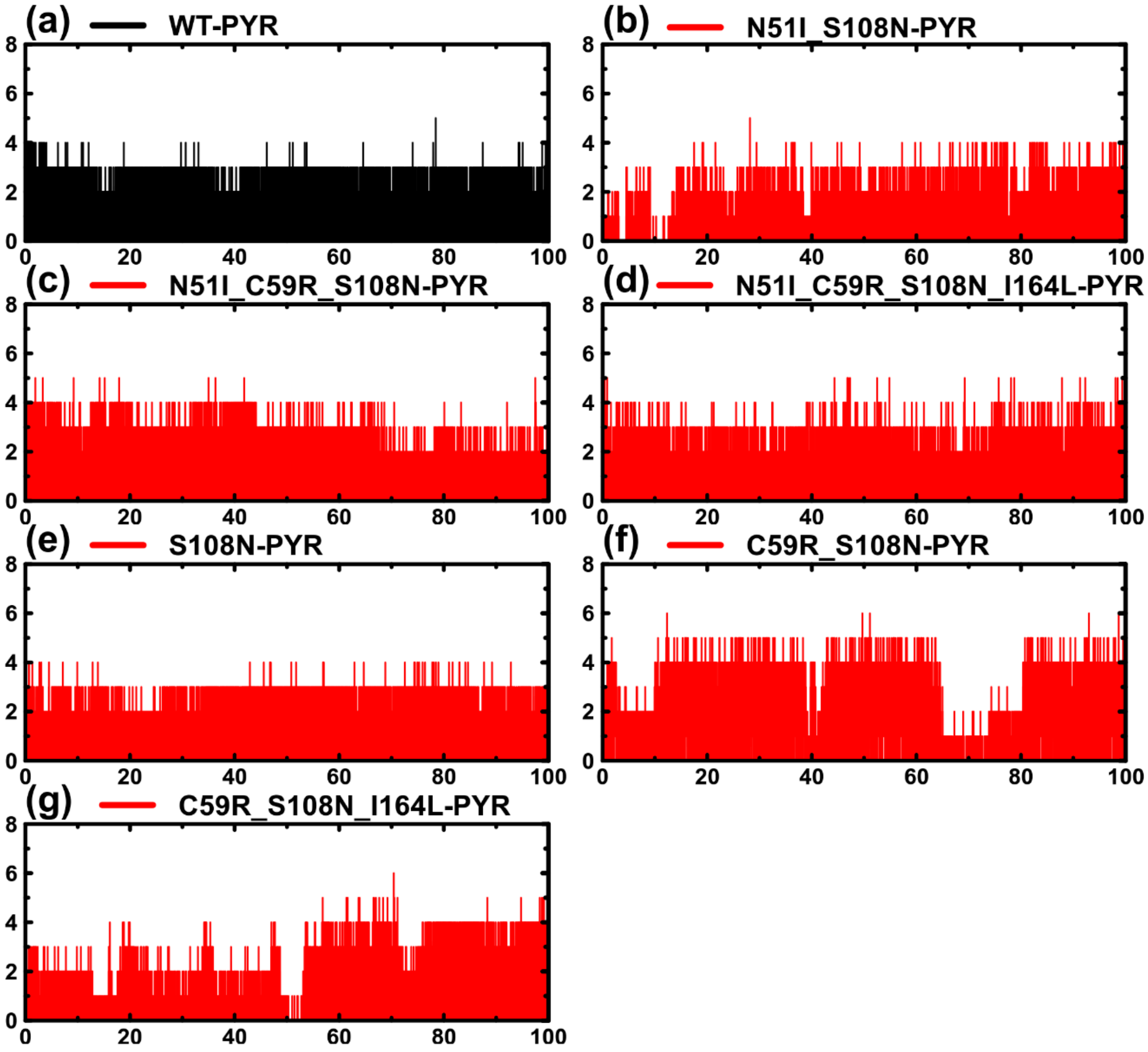

Figure S8: Hydrogen bond numbers yielded during 100ns simulation

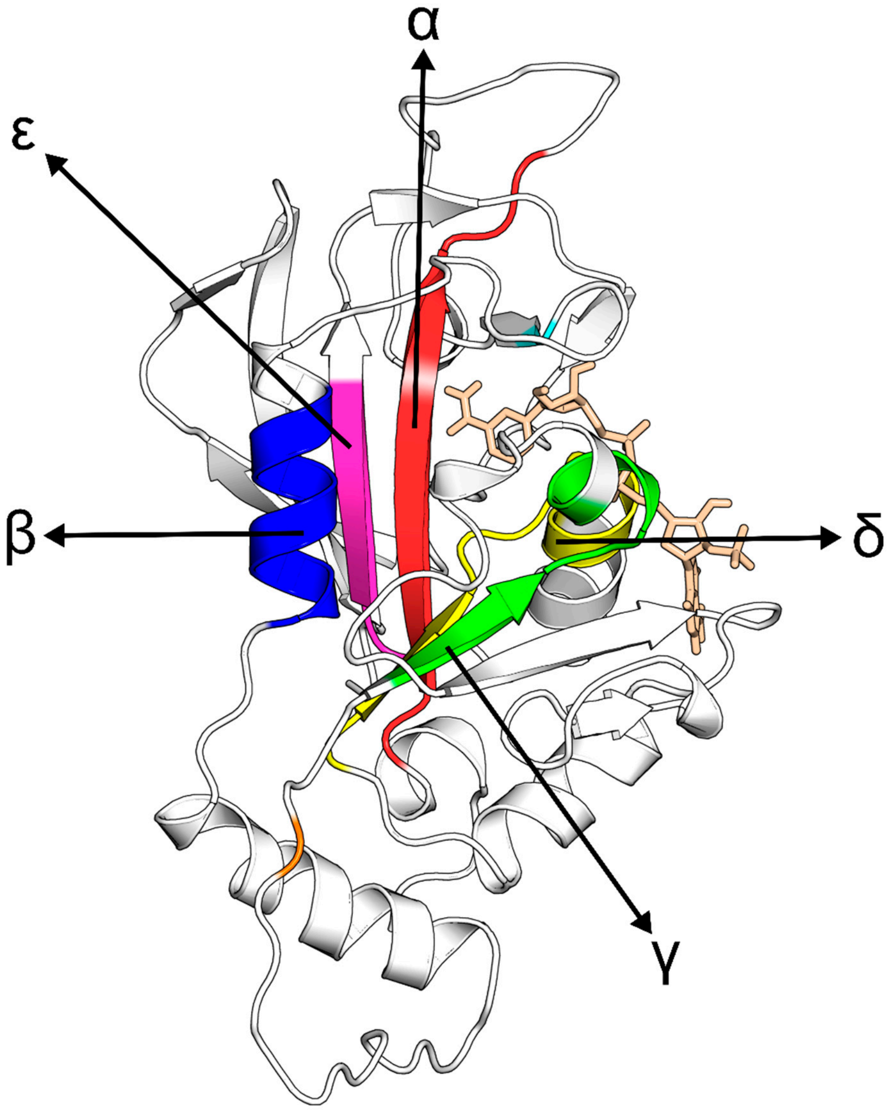

Figure S9: DHFR structure with mapped communication hubs (High *BC* centres). Different hubs are shown on separate secondary structures and numbered in ascending order based on residue numbering: 1:10-21, 2:55-63, 3:101-109, 4:159-170, 5:180-185. 41 and 196 are also mapped and do not belong to any of the hubs.

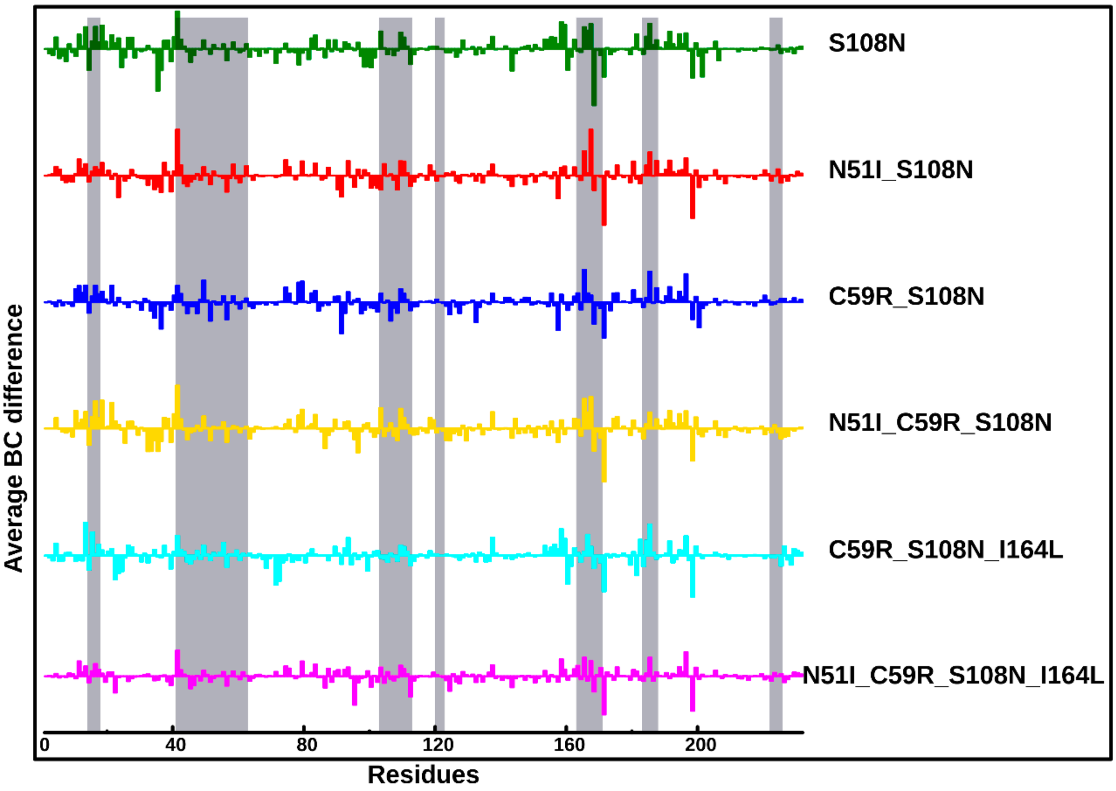

Figure S10. Effect of mutation on residue centrality (WT-free less mutant–free): Changes in residue betweenness centrality (average *BC* differences(ΔBC)) were obtained from calculations of WT-free less mutant–free values. Upward facing bars represent decrease in average *BC* for mutant–free relative to WT–free systems and vice versa for downward facing bars. Height and depth of bars represent magnitude of change. Shaded areas are regions of ligand interaction within the active site.

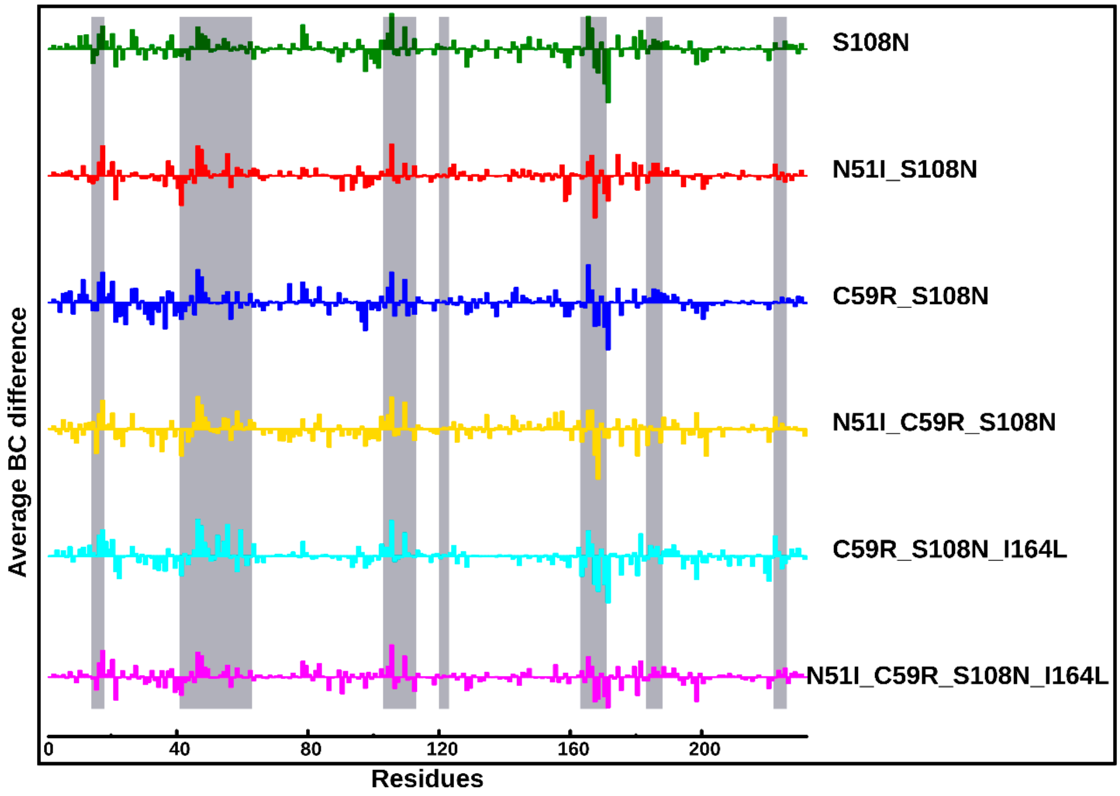

Figure S11. Effect of mutation on residue centrality (WT-bound less mutant–bound): Changes in residue centrality (average *BC* differences(ΔBC)) were obtained from calculations of WT-bound less mutant–bound values. Plotting scheme similar to figure S10 was applied. Shaded areas are regions of ligand interaction within the active site.

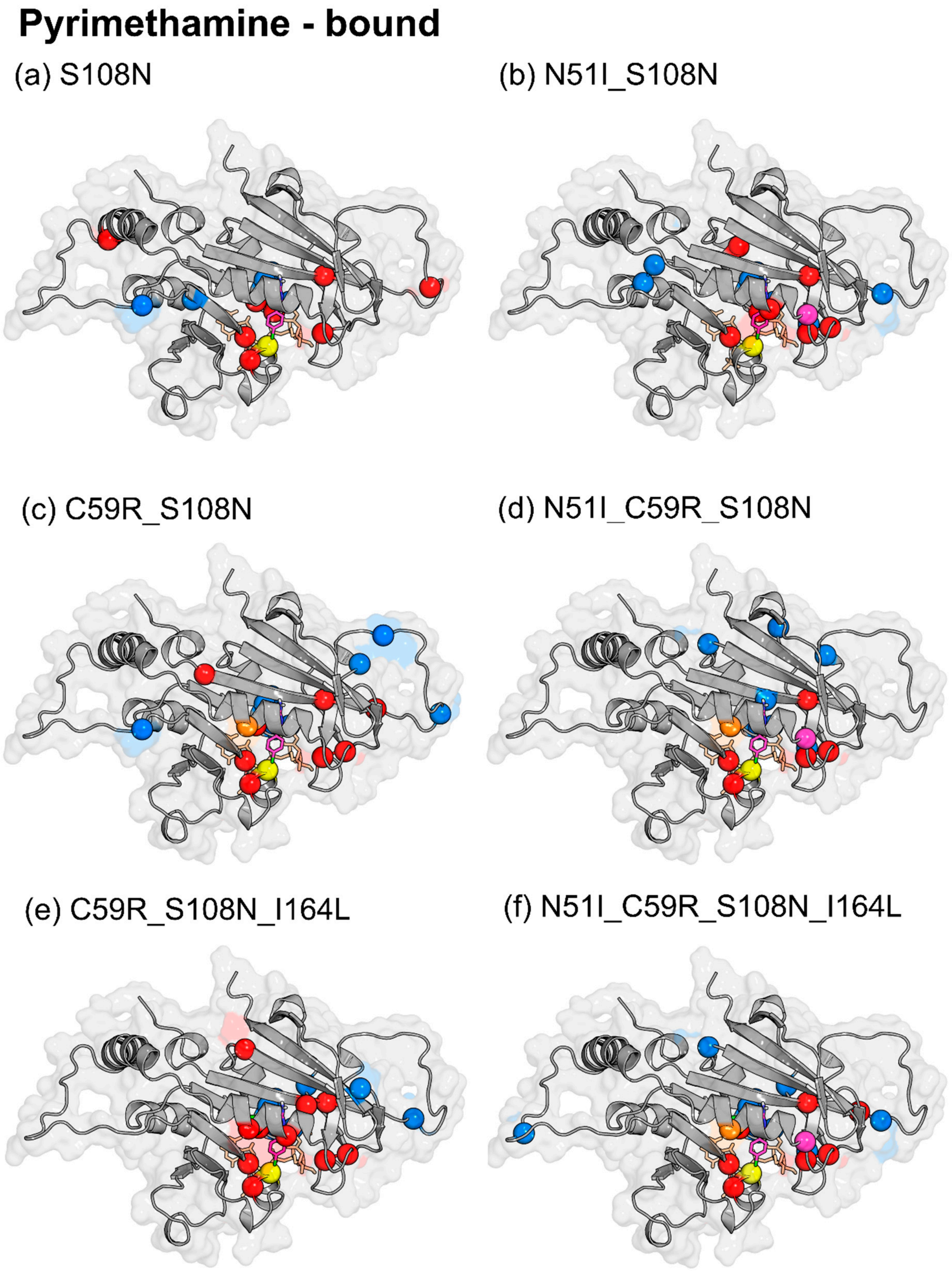

Figure S12: Structural mapping of residues that yielded large changes in average *BC* values (WT-PYR less Mutant-PYR) for pyrimethamine-bound *pf*DHFR models.

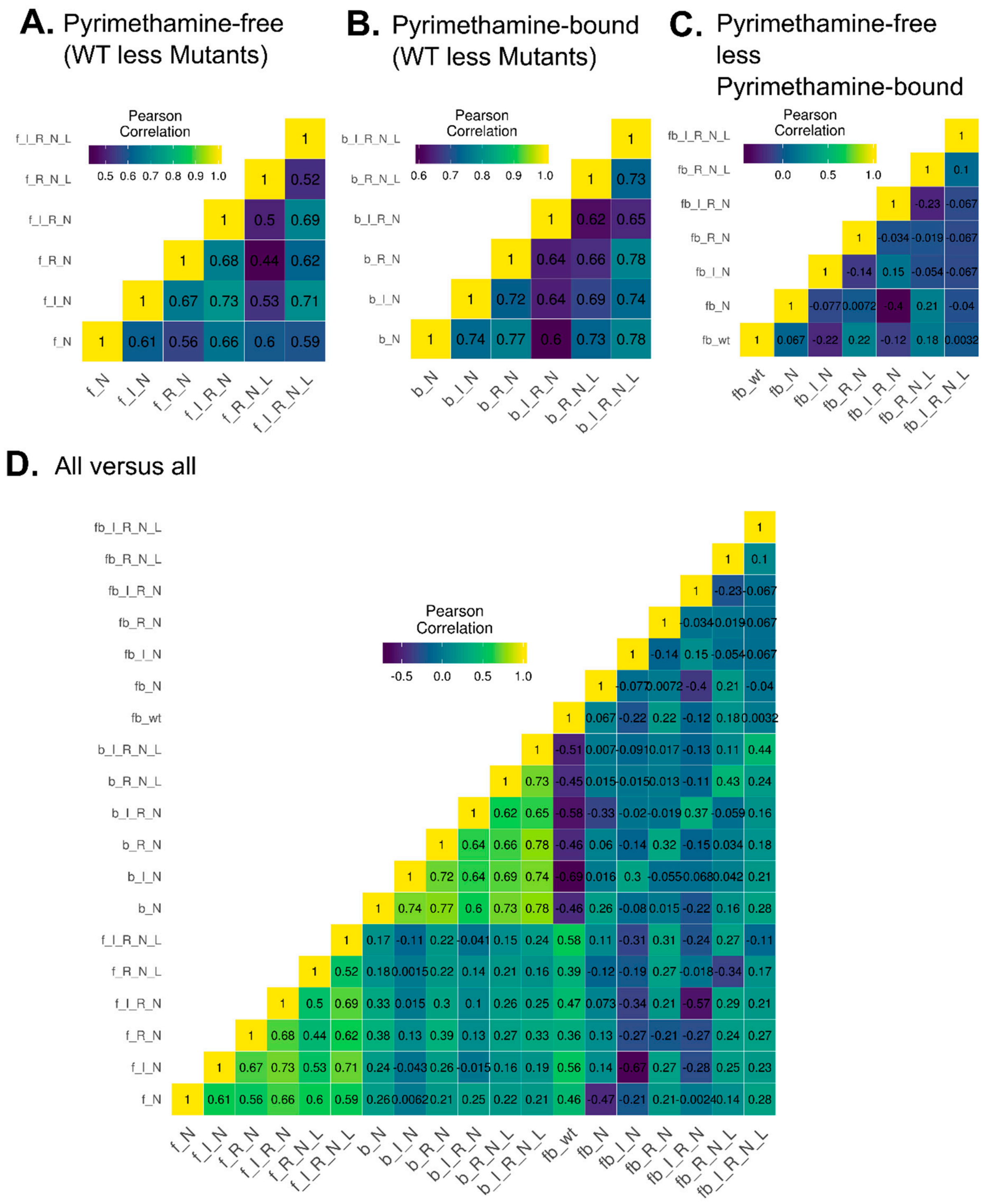

Figure S13: Pairwise Pearson's correlation heatmap of average BC differences(ΔBC): Key: N: S108N, I_N: N51I_S108N, R_N: C59R_S108N, I_R_N: N51I_ C59R_S108N, R_N_L: C59R_S108N_I164L, I_R_N_L: N51I_ C59R_S108N_I164L. The precursors f, b, and fb represent systems A: Pyrimethamine free systems (WT less mutants), B: Pyrimethamine-bound systems (WT- pyrimethamine less mutants- pyrimethamine), and C: Pyrimethamine-free less pyrimethamine-bound systems respectively.

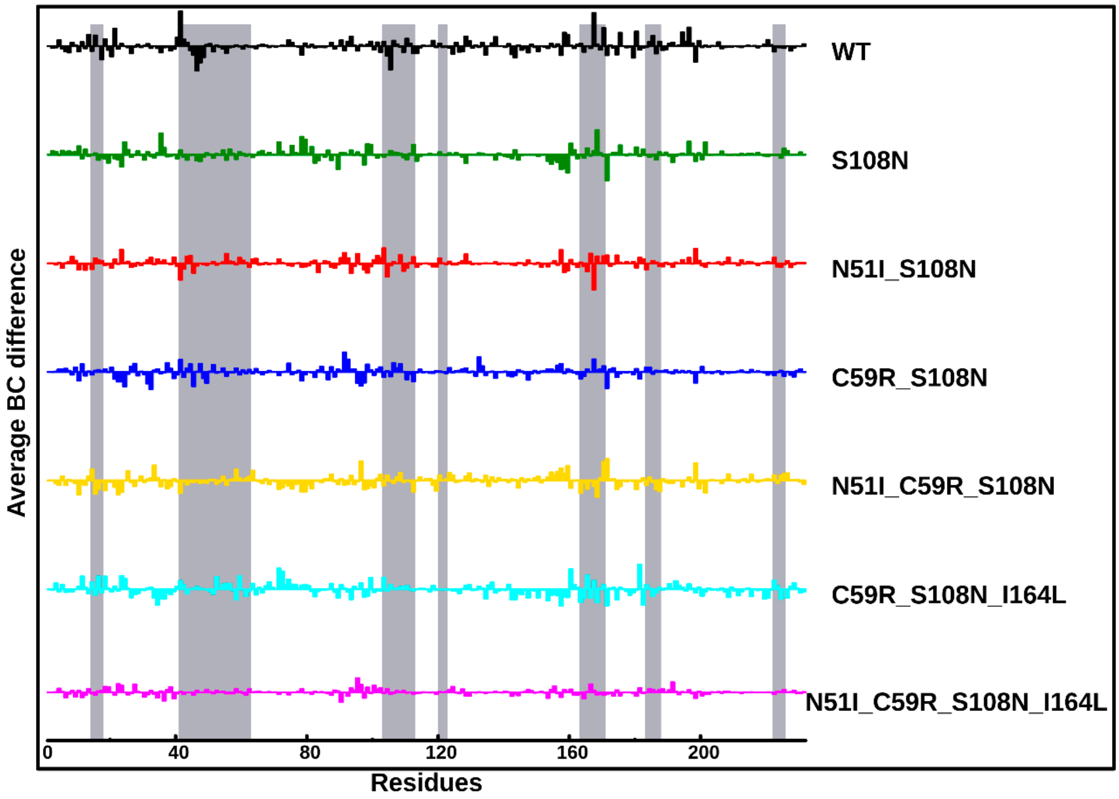

Figure S14: Effects of ligand binding on residue centrality (pyrimethamine-free less pyrimethamine-bound). Effects of ligand binding on residue centrality. Changes in residue centrality (average BC differences (ΔBC)) were obtained from calculations of pyrimethamine-free less pyrimethamine-bound values. Upward facing bars represent decrease in *BC* for pyrimethamine–bound relative to pyrimethamine–free system and vice versa for downward facing bars. Height and depth of bars represent magnitude of change. Shaded areas are regions of ligand interaction within the active site.

Table S7: Summary of equilibrated trajectory regions sampled for analyses of binding free energy, and dynamic residue interaction network (DRN).

	System	Time (ns)
Pyrimethamine-bound	Wildtype – PYR	85 - 100
	S108N – PYR	85 - 100
	N51I_S108N – PYR	85 - 100
	C59R_S108N – PYR	85 - 100
	N51I_ C59R_S108N – PYR	85 - 100
	C59R_S108N_I164L – PYR	85 - 100
	N51I_ C59R_S108N_I164L – PYR	85 - 100
Pyrimethamine-free	Wildtype	70 – 85
	S108N	85 - 100
	N51I_S108N	85 - 100
	C59R_S108N	85 - 100
	N51I_ C59R_S108N	85 - 100
	C59R_S108N_I164L	85 - 100
	N51I_C59R_S108N_I164L	85 - 100
