# Supplementary figures and images for: Understanding the Pyrimethamine Drug Resistance Mechanism via Combined Molecular Dynamics and Dynamic Residue Network Analysis
Source: Molecules. 2020 Feb 18;25(4):904. doi: 10.3390/molecules25040904 (PMC7070769; doi:10.3390/molecules25040904)

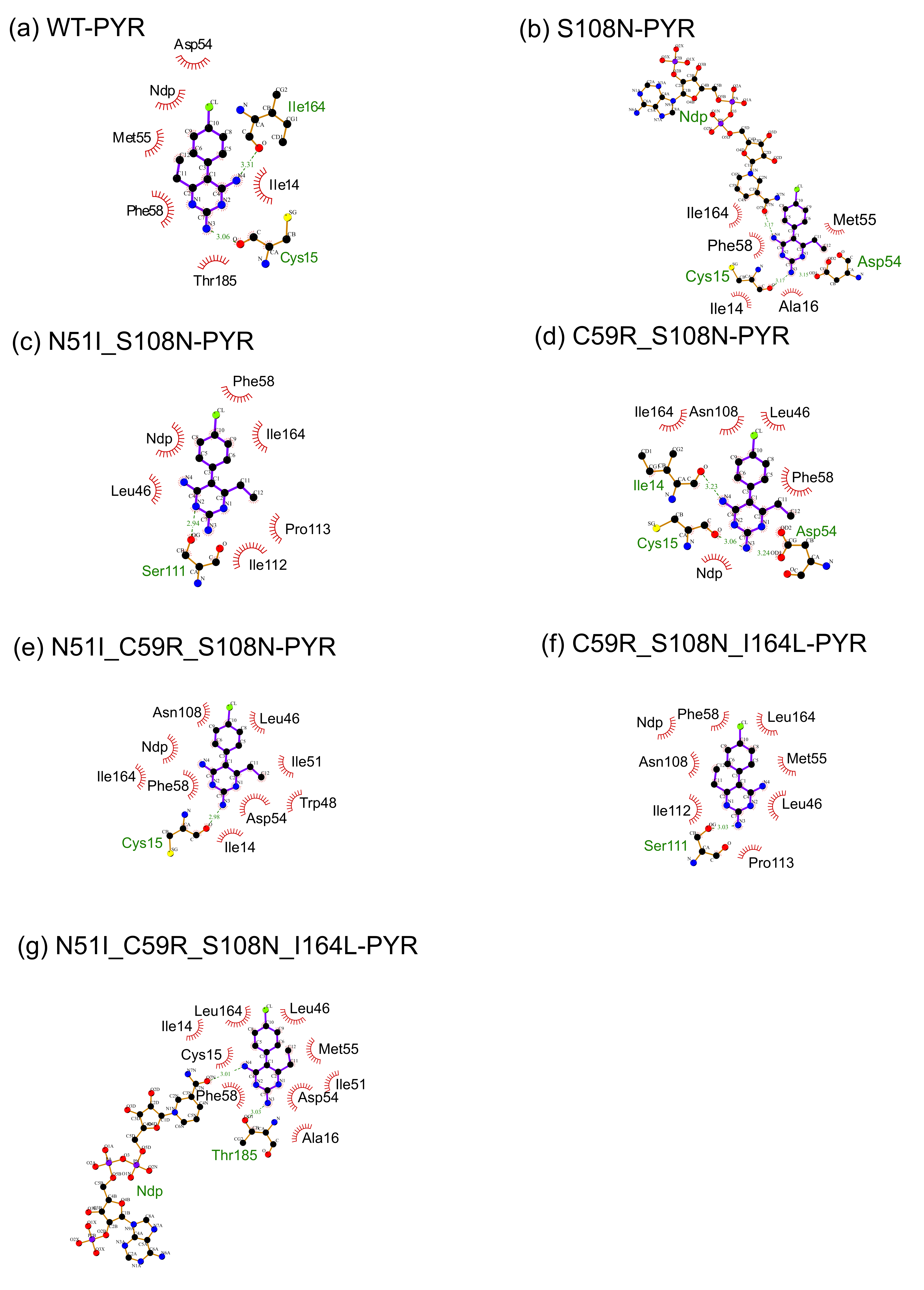

Supplement: Supplementary file 1 [file molecules-25-00904-s001.zip › molecules-676872-SI/Supplementary_Figures/Figure_S1.tiff]

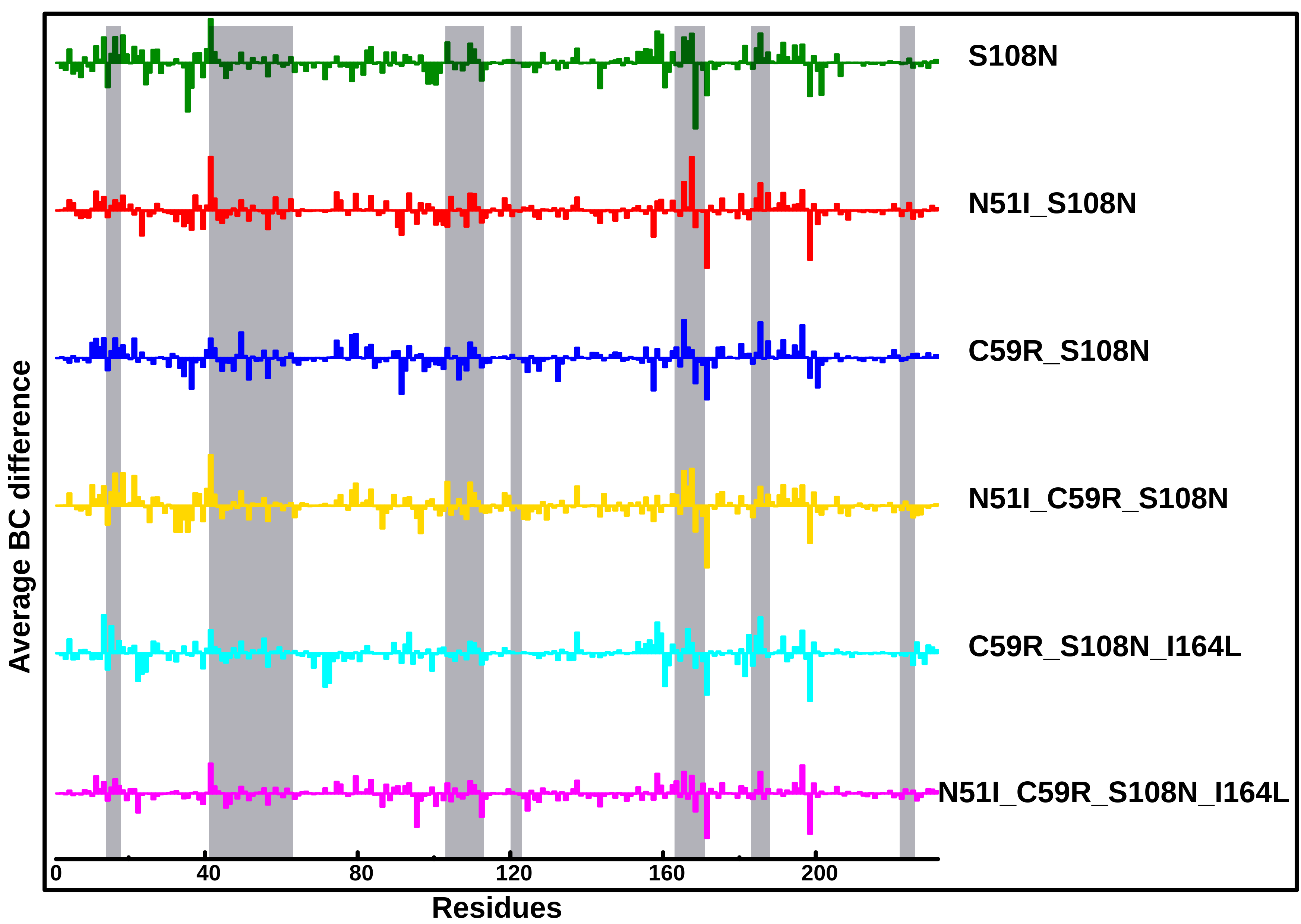

Supplement: Supplementary file 1 [file molecules-25-00904-s001.zip › molecules-676872-SI/Supplementary_Figures/Figure_S10.tiff]

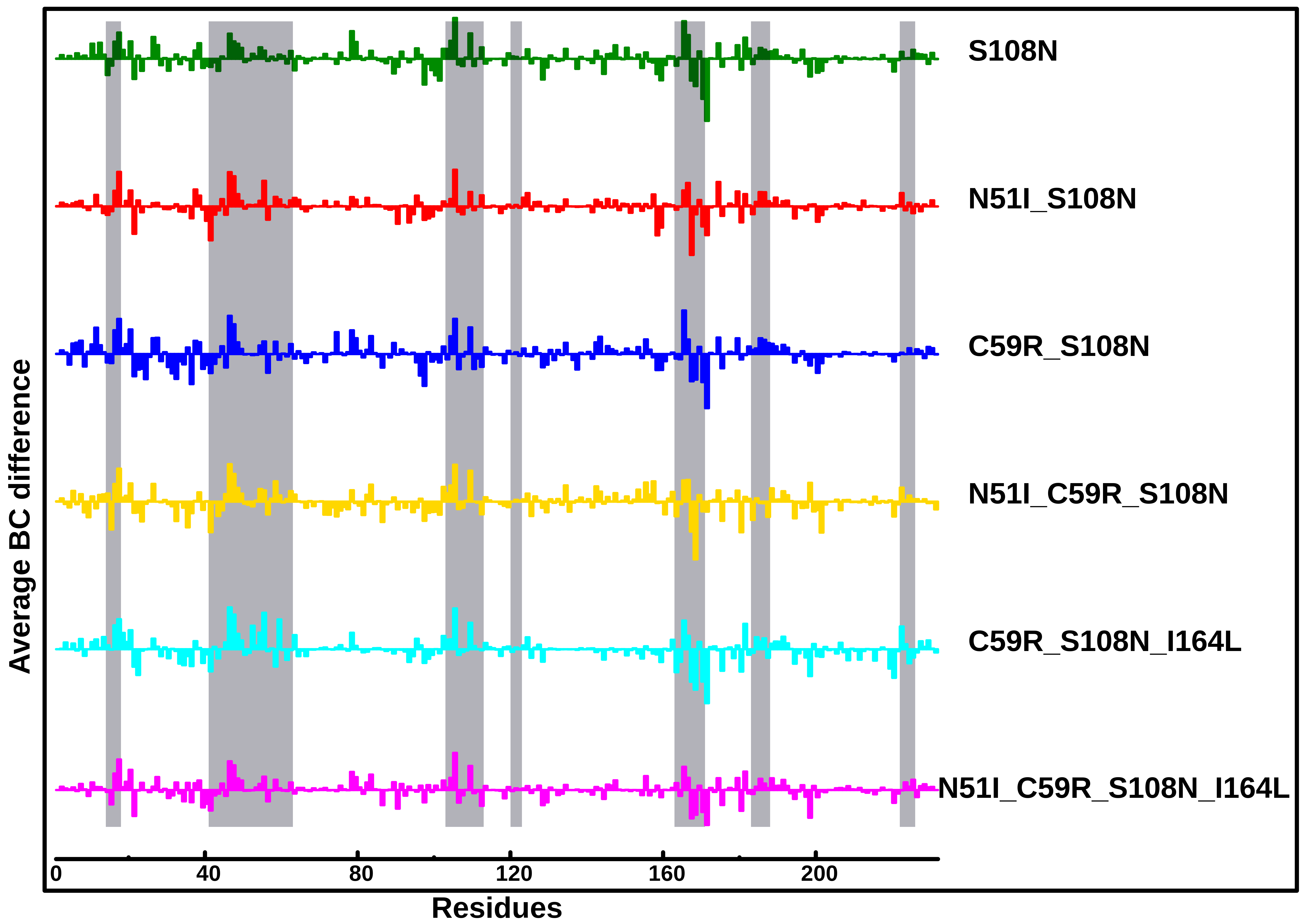

Supplement: Supplementary file 1 [file molecules-25-00904-s001.zip › molecules-676872-SI/Supplementary_Figures/Figure_S11.tiff]

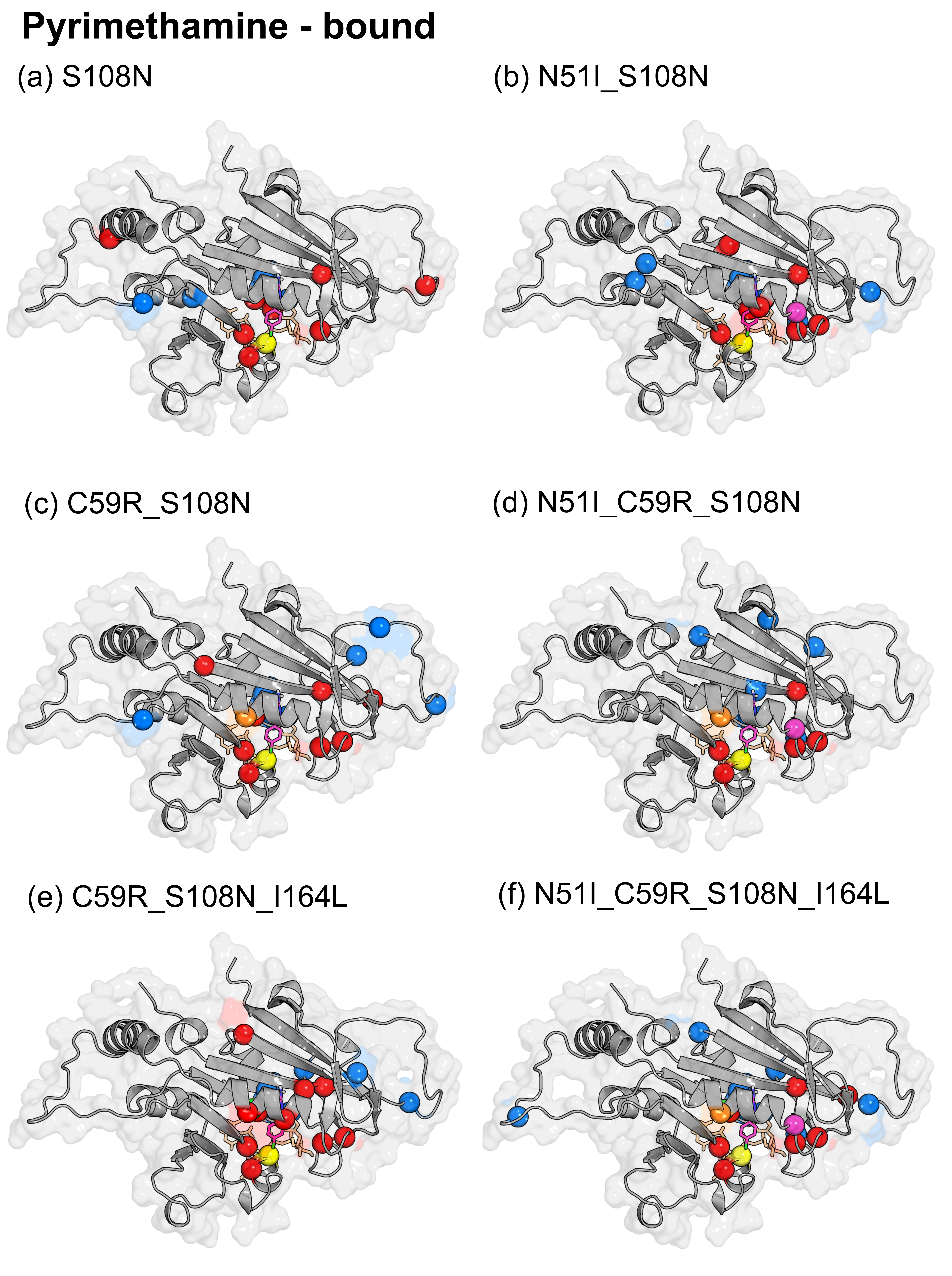

Supplement: Supplementary file 1 [file molecules-25-00904-s001.zip › molecules-676872-SI/Supplementary_Figures/Figure_S12.tiff]

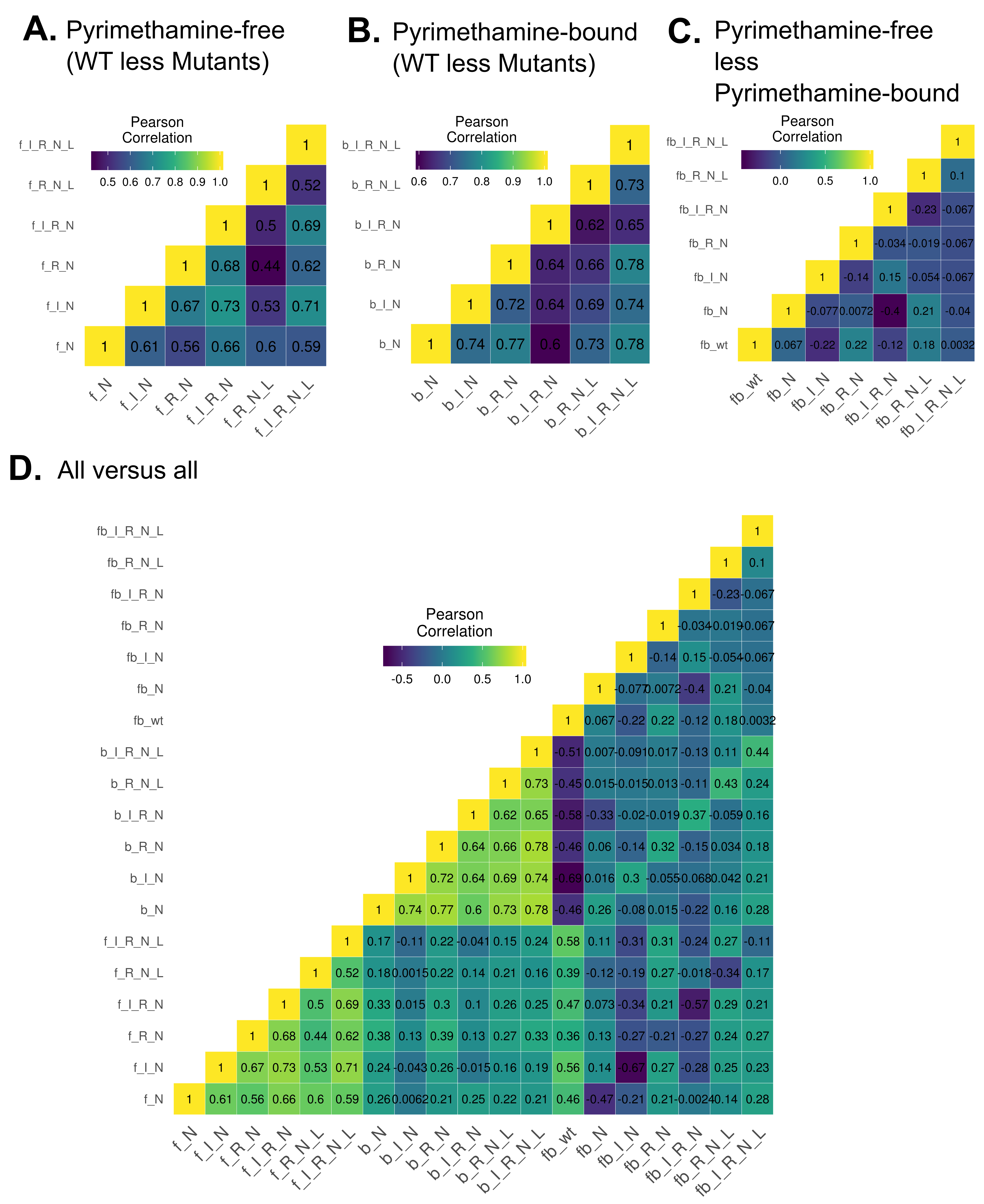

Supplement: Supplementary file 1 [file molecules-25-00904-s001.zip › molecules-676872-SI/Supplementary_Figures/Figure_S13.png]

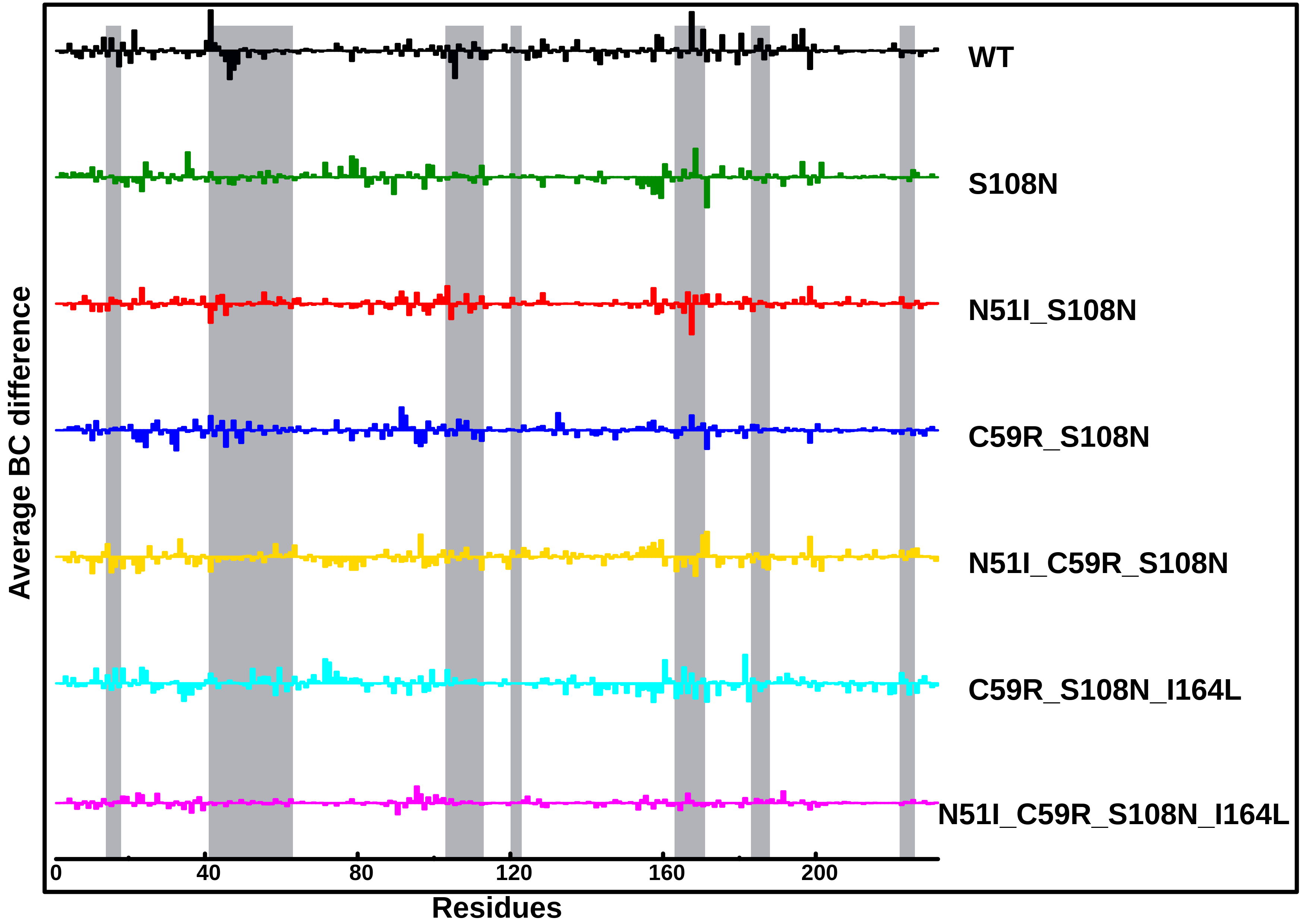

Supplement: Supplementary file 1 [file molecules-25-00904-s001.zip › molecules-676872-SI/Supplementary_Figures/Figure_S14.tiff]

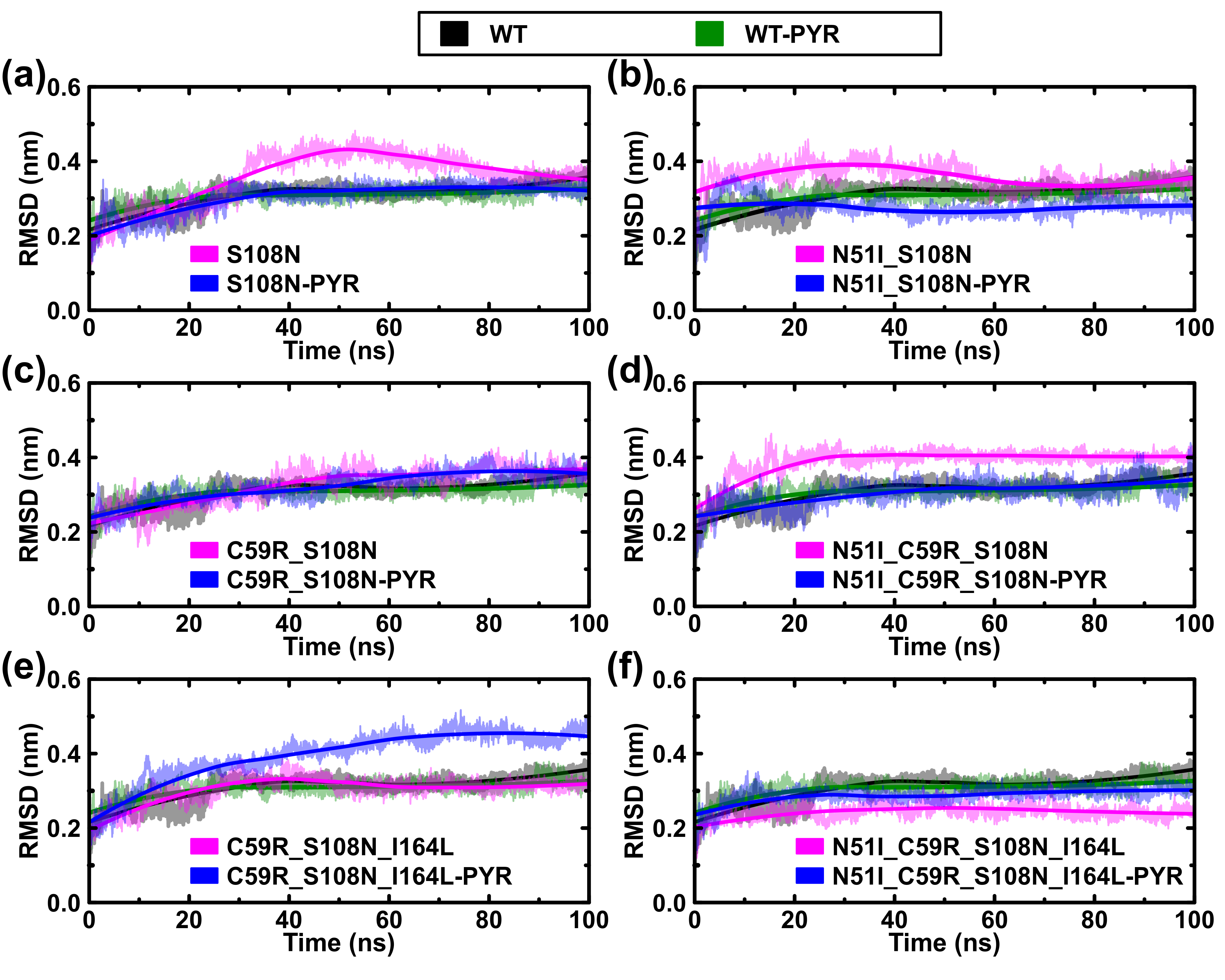

Supplement: Supplementary file 1 [file molecules-25-00904-s001.zip › molecules-676872-SI/Supplementary_Figures/Figure_S2.tiff]

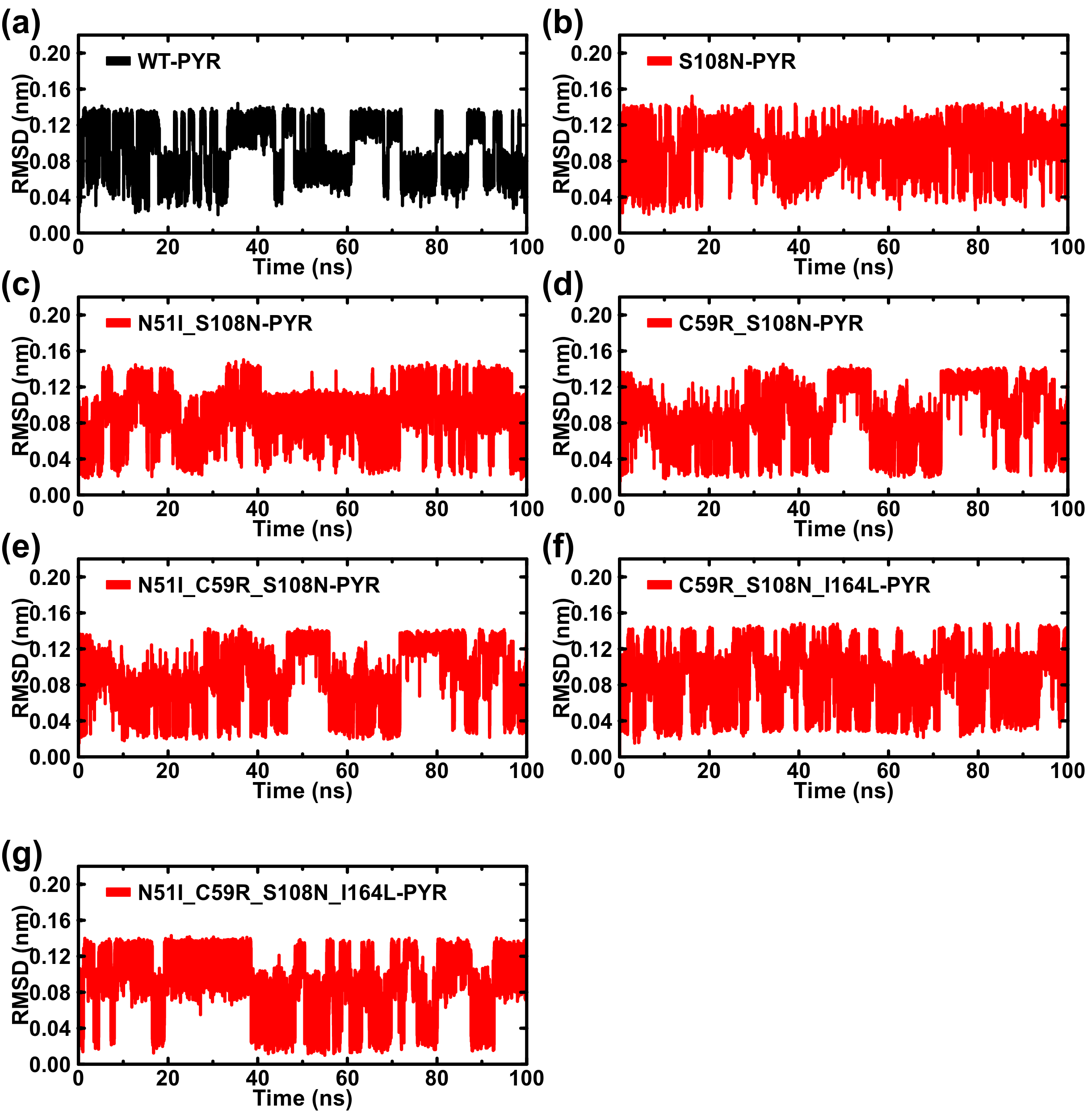

Supplement: Supplementary file 1 [file molecules-25-00904-s001.zip › molecules-676872-SI/Supplementary_Figures/Figure_S3.tiff]

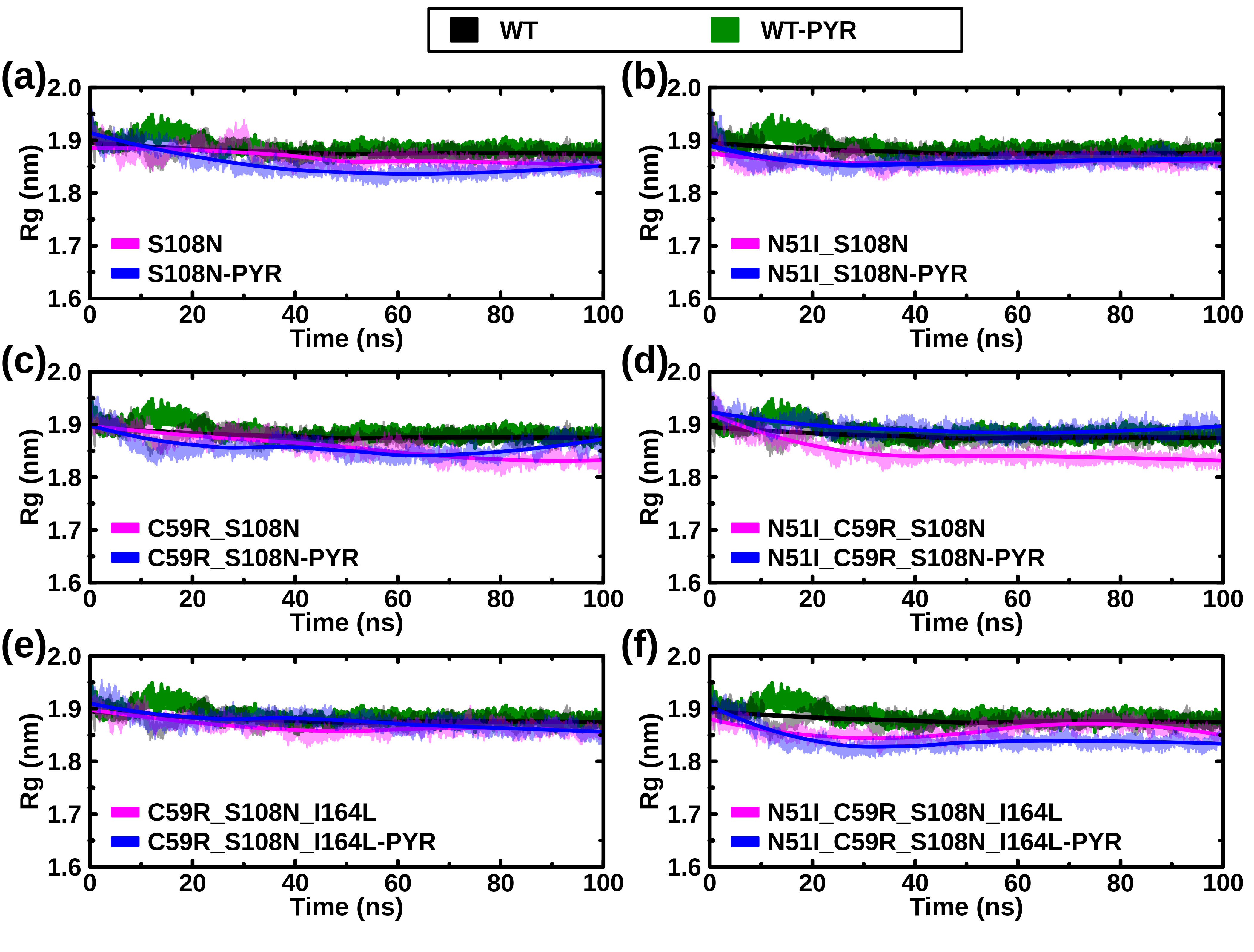

Supplement: Supplementary file 1 [file molecules-25-00904-s001.zip › molecules-676872-SI/Supplementary_Figures/Figure_S4.tiff]

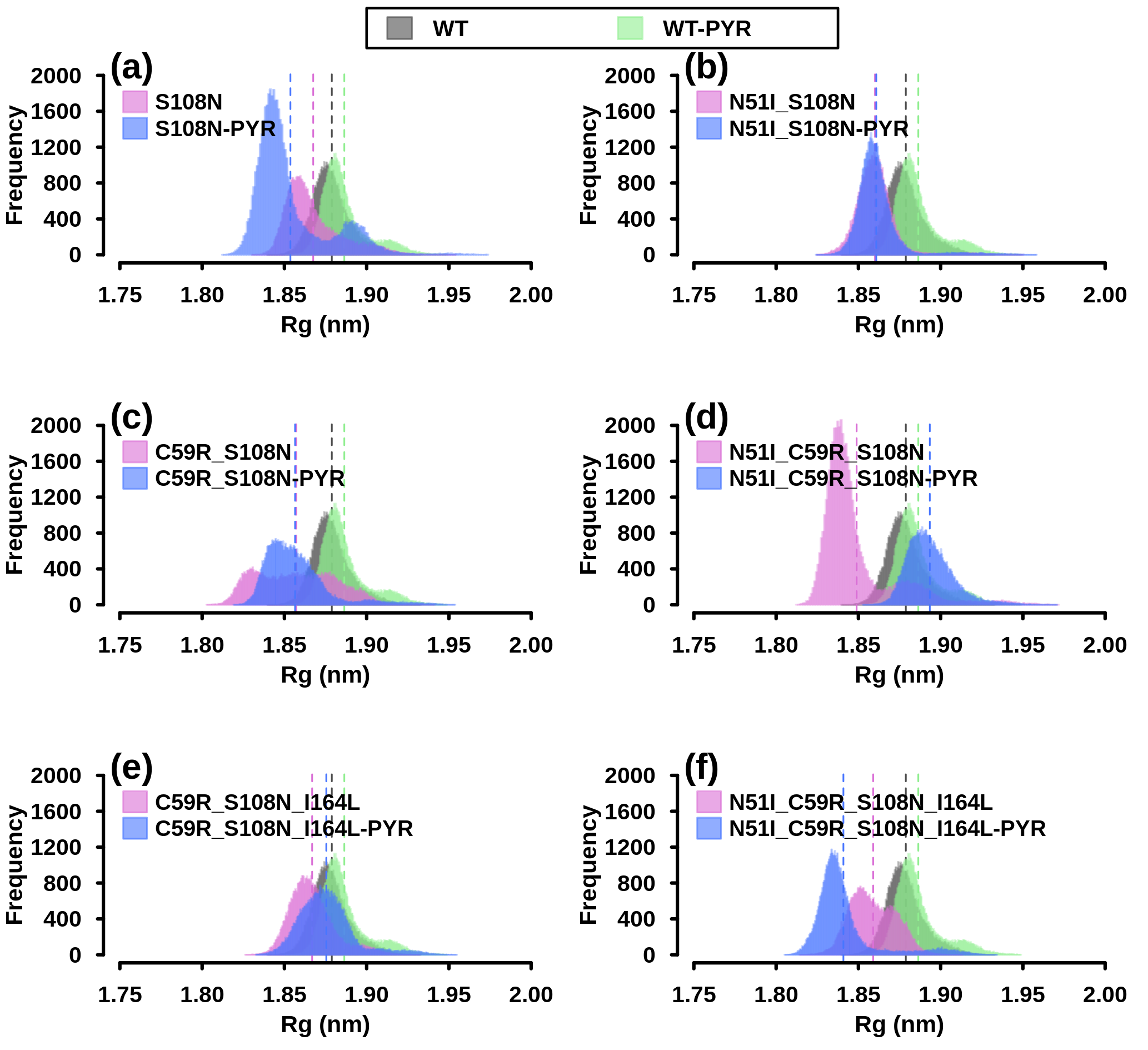

Supplement: Supplementary file 1 [file molecules-25-00904-s001.zip › molecules-676872-SI/Supplementary_Figures/Figure_S5.tiff]

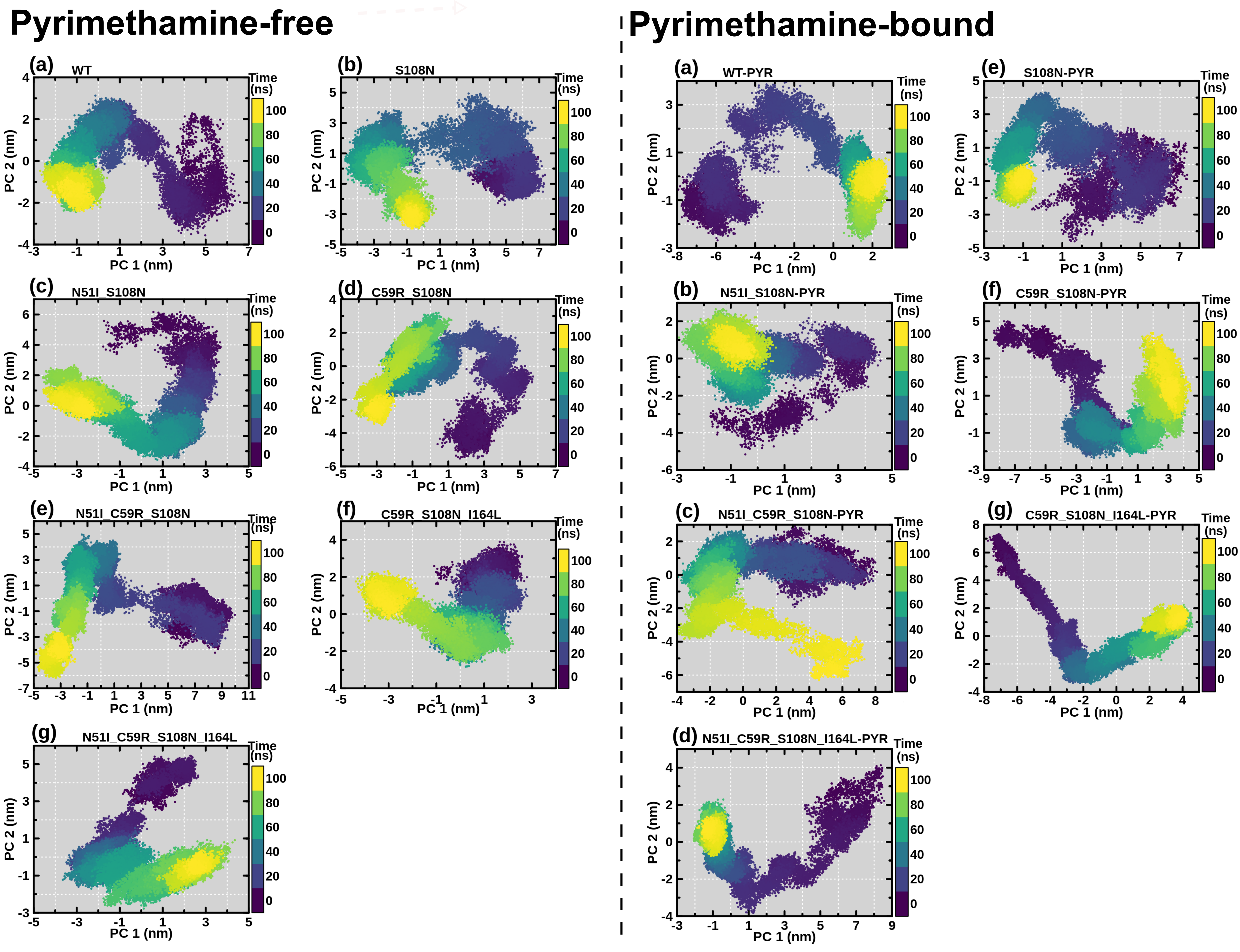

Supplement: Supplementary file 1 [file molecules-25-00904-s001.zip › molecules-676872-SI/Supplementary_Figures/Figure_S6.png]

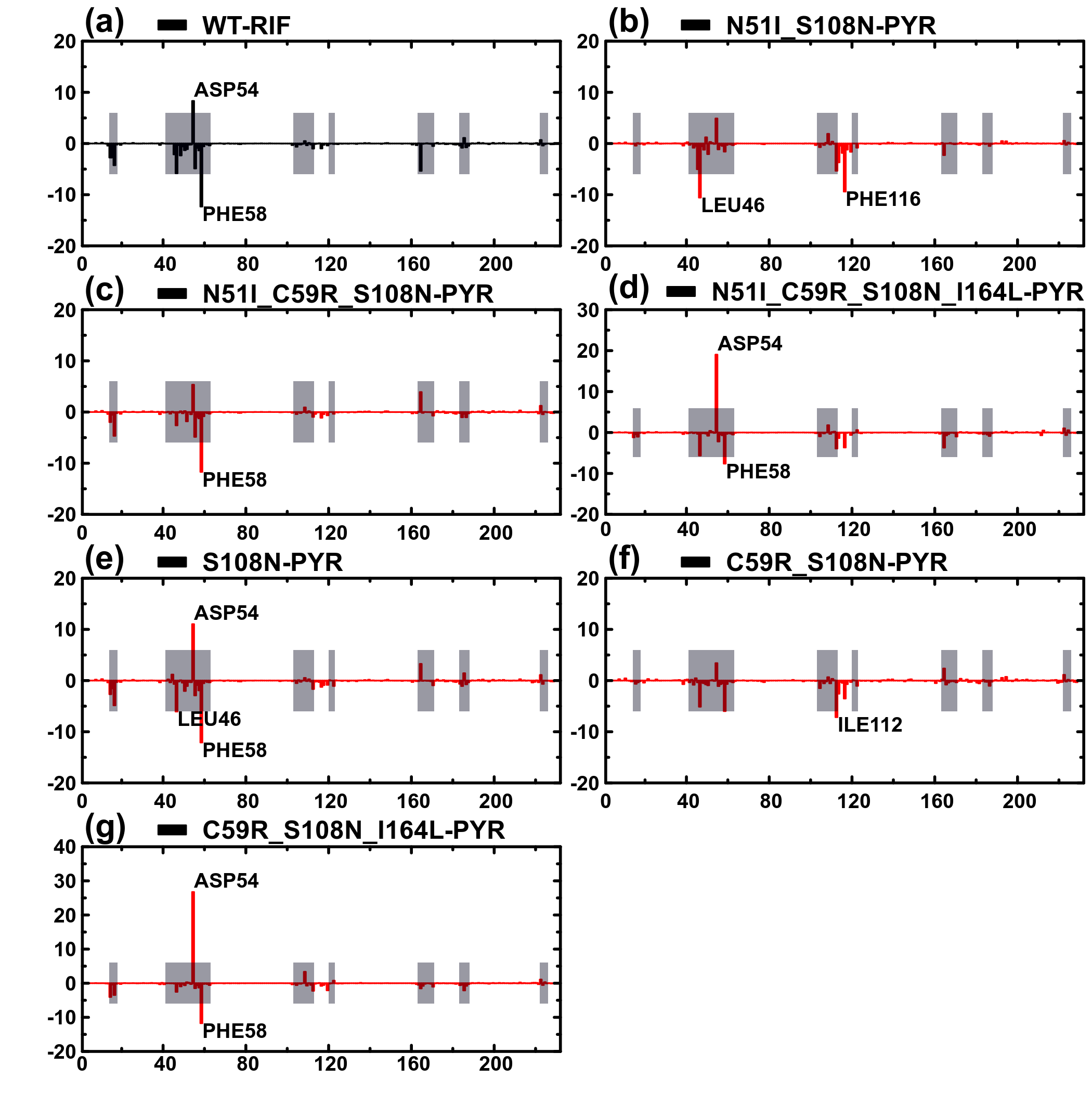

Supplement: Supplementary file 1 [file molecules-25-00904-s001.zip › molecules-676872-SI/Supplementary_Figures/Figure_S7.tiff]

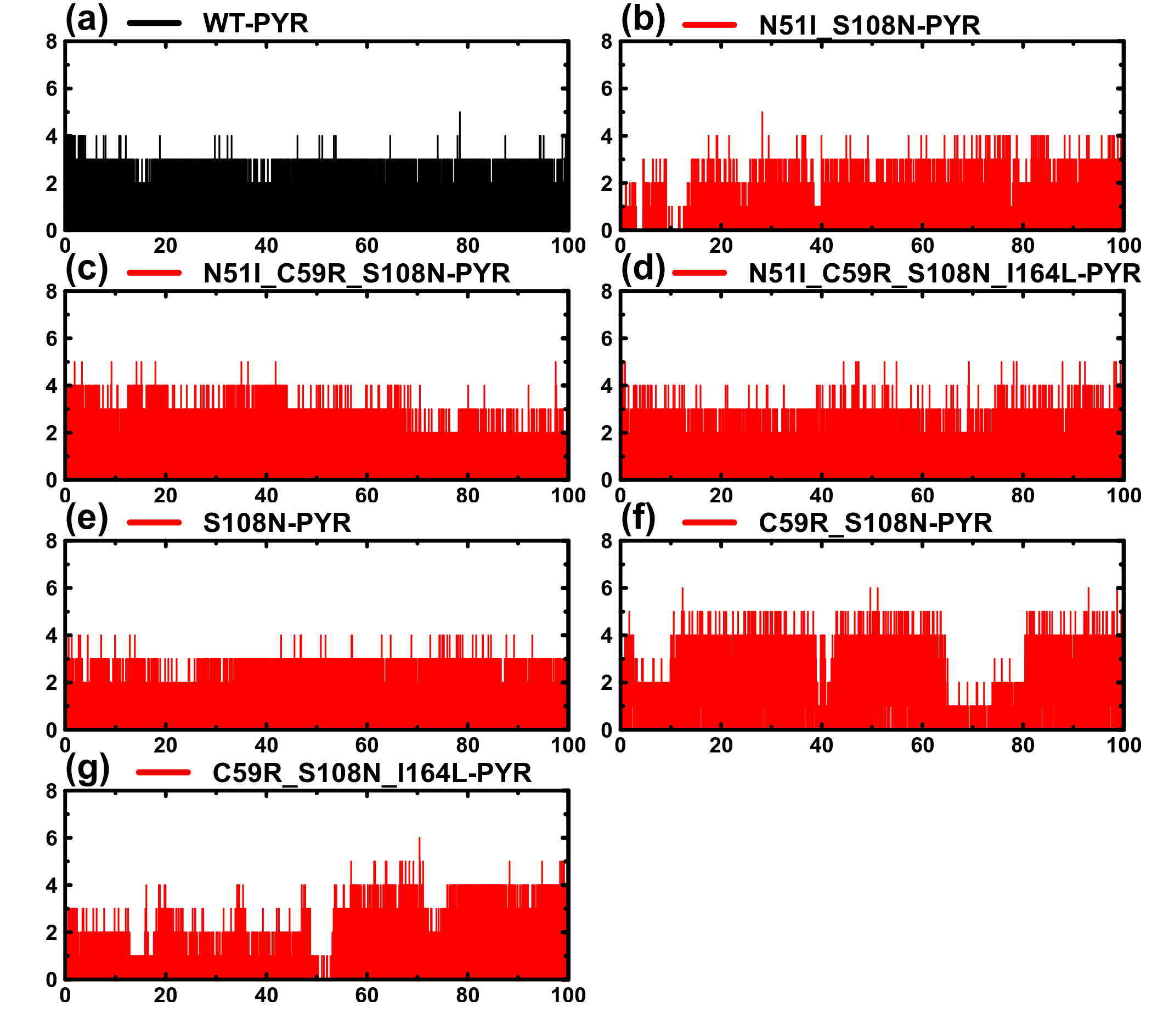

Supplement: Supplementary file 1 [file molecules-25-00904-s001.zip › molecules-676872-SI/Supplementary_Figures/Figure_S8.tiff]

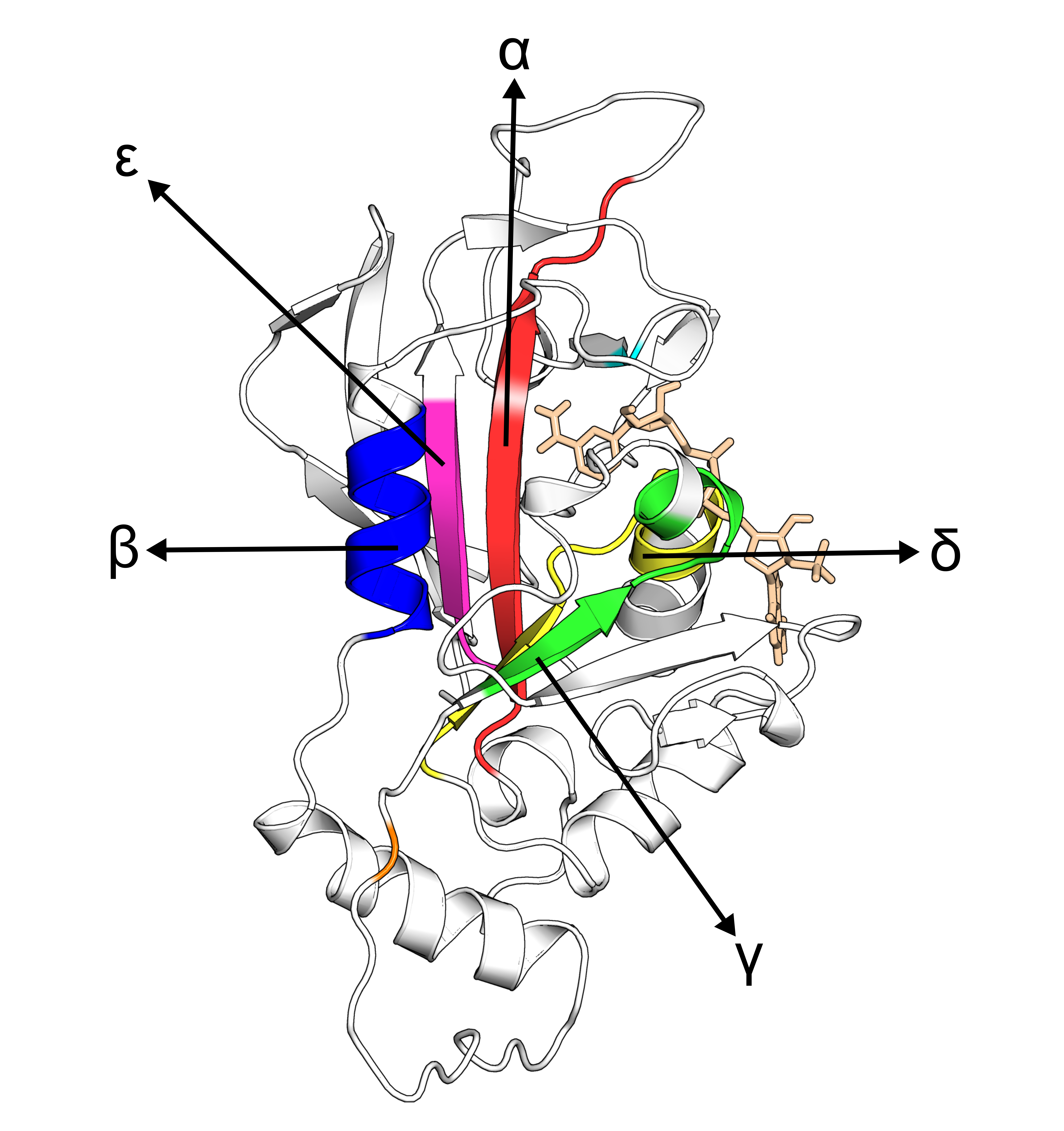

Supplement: Supplementary file 1 [file molecules-25-00904-s001.zip › molecules-676872-SI/Supplementary_Figures/Figure_S9.png]
